# Vapor-Deposited Thin Films: Studying Crystallization and α-relaxation Dynamics of the Molecular Drug Celecoxib

**DOI:** 10.1021/acs.jpcb.2c01284

**Published:** 2022-05-17

**Authors:** Aparna Beena Unni, Roksana Winkler, Daniel Marques Duarte, Wenkang Tu, Katarzyna Chat, Karolina Adrjanowicz

**Affiliations:** † Institute of Physics, University of Silesia, 75 Pulku Piechoty 1, 41-500 Chorzow, Poland; ‡ Silesian Center for Education and Interdisciplinary Research (SMCEBI), 75 Pulku Piechoty 1a, 41-500 Chorzow, Poland

## Abstract

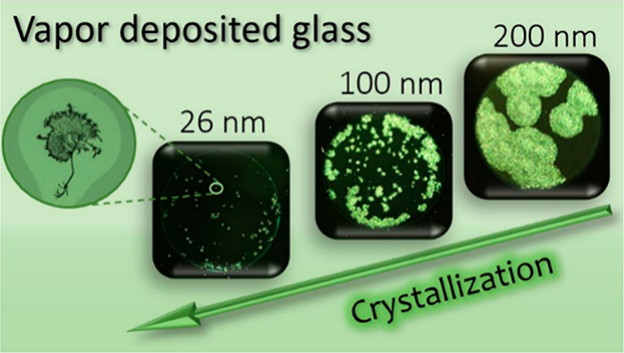

Crystallization is one of the major challenges in using glassy solids for technological applications. Considering pharmaceutical drugs, maintaining a stable amorphous form is highly desirable for improved solubility. Glasses prepared by the physical vapor deposition technique got attention because they possess very high stability, taking thousands of years for an ordinary glass to achieve. In this work, we have investigated the effect of reducing film thickness on the α-relaxation dynamics and crystallization tendency of vapor-deposited films of celecoxib (CXB), a pharmaceutical substance. We have scrutinized its crystallization behavior above and below the glass-transition temperature (*T*_g_). Even though vapor deposition of CXB cannot inhibit crystallization completely, we found a significant decrease in the crystallization rate with decreasing film thickness. Finally, we have observed striking differences in relaxation dynamics of vapor-deposited thin films above the *T*_g_ compared to spin-coated counterparts of the same thickness.

## Introduction

Molecular glasses are promising materials for many technological applications in pharmaceutics, organic electronics, optics, and so forth. They can have solid-like mechanical properties even though they are liquid-like and locally disordered. This local disorder and liquid-like nature can provide macroscopic homogeneity and compositional flexibility to glassy materials.^1^ Despite the enormous amount of work done in the past few decades, a detailed understanding of the glass formation and its properties remains a puzzle. One of the major challenges while using glasses for commercial application is that they undergo crystallization due to higher Gibbs free energy.^2−4^ Physical instability is the most significant problem related to amorphous pharmaceuticals.^5,6^ Considering our tested material, celecoxib (CXB), the amorphous state undergoes recrystallization very quickly over time when maintained at room temperature.^7−9^

The cutting-edge work by Ediger et al. has demonstrated that remarkably stable glasses can be produced by using the physical vapor deposition (PVD) technique.^10,11^ This technique is reported to bypass the kinetic restrictions and produce glassy materials with impressive properties, including very high thermal and kinetic stability, photostability, orientational and translational structure order, and density.^10−18^ Compared to ordinary glass produced by rapid cooling, the structural relaxation time of vapor-deposited glass can take thousands of years. Thus, they are generally termed “ultrastable” or “superaged” glasses. When prepared under specific deposition conditions, vapor-deposited glasses can attain near-equilibrium packing. The key to understanding the formation of such extraordinarily stable glasses is enhanced surface mobility, which is 10^6^ to 10^8^ times faster than bulk mobility.^19−21^ Enhanced mobility at the interface allows the molecules to efficiently explore the configuration space and thus find a lower position on the energy landscape to approach a closer equilibrium configuration.^22,23^ The recent work by Samanta et al. showed that a molecule with no enhanced surface diffusion could form an ultrastable glass.^24^ It is also important to mention that Zhang and Fakhraai have reported that the surface diffusion and relaxation dynamics are indeed decoupled.^25^ A more detailed discussion on the factors affecting the ultrastability has recently been published by Rodríguez-Tinoco et al.^26^

Moving to the properties of vapor-deposited glasses, they are known to be substantially influenced by processing conditions. For instance, the rate of material deposition affects the structural anisotropy, thermal stability, and density of the vapor-deposited glass.^22,27,28^ Likewise, the temperature of the substrate (*T*_sub_) during deposition can modify the surface equilibration kinetics, stability, molecular orientation, and many others.^11,16,29−35^ Focusing on the deposition rate, especially around 0.5 nm s^–1^, the molecules are said to obtain enough time for the configurational sampling, allowing high density and stability. Whereas increasing the substrate temperature during deposition increases the surface mobility, which is essential for better packing. Generally, the optimum deposition temperature is around ∼0.85*T*_g_ for most glass-formers, where *T*_g_ is the glass-transition temperature of the material. Above *T*_g_, the equilibration is too fast to obtain highly dense ultrastable glasses. Recently, a deposition rate-substrate temperature superposition rule was established for vapor-deposited glasses. From a change in the substrate temperature that results in alteration of a given property by a certain amount, one can predict the change in deposition rate necessary to modify the considered property by the same amount.^36^

In recent years, there has been a growing interest in studying the properties of vapor-deposited glasses under nanoscale confinement. The first experimental evidence of the size effect on the glass transition of vapor-deposited glasses was put forward by Leon-Gutierrez et al.^37^ For vapor-deposited toluene, it was found that the fictive temperature and the onset temperature of the thinnest films were significantly reduced compared to the bulk. In contrast, the ordinary glass thin films down to 2 nm thickness, obtained by deposition at a high rate, did not show any significant variation in the *T*_g_ due to confinement.^38^ The AC nano-calorimetry studies on confined vapor-deposited films have shown a thickness-dependent transformation time to form a supercooled liquid for films thinner than 1 μm.^39^ They found that the relaxation mechanism is significantly affected by the film thickness. The confined glasses deposited at faster rates (∼15 nm s^–1^) were also found to possess an increased transformation rate.^40^ A recent study by Jin et al. demonstrated that the high density-supercooled liquid state is thermodynamically favored only in vapor-deposited glasses with the film thickness of 25 to 55 nm.^41^ When it comes to OLED devices, the surface potential depends on the film thickness, especially for polar organic semiconductors.^42^ It has also been demonstrated that interfacial molecular packing in vapor-deposited films of organic semiconductors as thin as ∼13 nm is more disordered than in bulk.^43^ Hence, it is undoubted that there is a significant influence of confinement on the properties of vapor-deposited glasses.

In this work, we study the α-relaxation dynamics and the crystallization behavior of vapor-deposited glass of pharmaceutical CXB under 1D confinement, mainly through dielectric spectroscopy (DS). Rodríguez-Tinoco et al. have reported the possibility of making ultrastable glasses of CXB by PVD. Vapor-deposited CXB glasses of micrometer size thickness possessed high thermal stability and were less susceptible to crystallization.^44^ In our case, we put forward a distinctive study to understand the properties of such films under 1D nanoconfinement. We have investigated the properties of vapor-deposited glasses with varying thicknesses down to 26 nm deposited at an optimal rate with the substrate temperature (*T*_sub_) at 0.85*T*_g_. We find that confinement by film thickness can significantly alter the molecular mobility associated with glass transition and the crystallization tendency of vapor-deposited glasses. Even though we do not expect differences in the structural dynamics between spin-coated (SC) and vapor-deposited materials of the same thickness when warmed up above the glass-transition temperature, interestingly, we found striking differences in the evolution of α-relaxation times for vapor-deposited and SC films of similar thicknesses above *T*_g_.

## Experimental Details

We use CXB with a molecular weight of 381 g/mol, supplied as a white crystalline powder by Polpharma (Starogard Gdanski, Poland). The molecular structure of CXB can be found in Figure S1 (Supporting Information). The melting point of the crystalline material (as received) determined from calorimetric studies is *T*_m_ = 435 K. The value of the glass-transition temperature recorded for the quenched-cooled sample upon a 10 K/min heating scan is *T*_g_ = 326.6 K. Both values, *T*_m_ and *T*_g_, agree with the literature data.^7^ The DSC thermograms for bulk CXB are given in Figure S2 (Supporting Information).

Conductive silicon wafers with a native oxide layer were used as the substrates (resistivity = 0.001–0.003 Ωcm, orientation (1 0 0)). Silicon wafers were supplied from Sil’Tronix (France). Before any deposition, the wafers were diced into pieces of dimension ∼15 × 15 mm^2^, purged using a nitrogen spray gun, and plasma-cleaned (Henniker Plasma HPT-100) to remove any possible organic contaminants from the surface.

PVD was performed in a custom-made vacuum system designed and assembled at the University of Silesia by MeasLine (Kraków, Poland). The details of vapor deposition are given in the Supporting Information. In the case of CXB, the crystalline material was loaded in an alumina crucible and heated inside the organic vacuum chamber. The substrate temperature was kept at 277 K (0.85 of the bulk *T*_g_) during deposition. The deposition rate was around 0.2 nm/s measured in situ during evaporation by the quartz crystal microbalance (QCM). The thickness of the deposited layer measured by QCM was compared with the atomic force microscopy (AFM) results. For the AFM experiment, we made a scratch on the CXB film surface using a soft pen and measured the height of the step using JPK’s NanoWizard 3 NanoScience AFM. The AFM measurements were achieved in a tapping mode using a silicon cantilever and analyzed using Gwyddion and WSxM software. The thickness of the obtained films was also confirmed by ellipsometry (Semilab SE2000 spectrometer). The measurements were obtained at incident angles of 65, 70, and 75° under ambient conditions. A multilayer model consisting of the Si substrate, native oxide layer, and CXB was considered. The average evaporation rate determined by QMB agrees with the rate calculated based on the film thickness and the time taken for the deposition process.

Nanometrically sized thin films of CXB were also prepared by SC. To do that, crystalline CXB was dissolved in methanol with various solution concentrations. Then, solutions were SC (KLM SCC-200) onto the surface of cleaned silicon wafers at 2000 rpm for 60 s to obtain the desired film thickness. This procedure allowed us to obtain homogeneous thin films. After preparation, thin films were annealed in a vacuum oven before further measurements. For the SC experiment, we use the same substrate material—conductive silicon wafers—as for PVD. Wafers were also cleaned following the same procedures. AFM and ellipsometry were used to verify film thicknesses.

Optical microscopy images were collected using an Olympus BX51 microscope equipped with a Moticam 3.0 camera. Images were taken in the reflection mode and analyzed using Motic Images Plus 2.0 ML software. The dielectric measurements were performed using a Novocontrol Alpha dielectric spectrometer within the frequency ranging from 10^–1^ to 10^6^ Hz. The temperature was controlled with stability better than 0.1 K by the Novocontrol Quatro system. For bulk CXB, the measurements were carried out after vitrification by fast cooling of the melt. The gap between the standard plate–plate electrodes was maintained using 20 μm thick Teflon strips, which act as spacers. The highly conductive silicon substrate on which CXB was vapor-deposited/SC acts as the lower electrode. Considering the thin-film dielectric measurements, a 1 × 1 mm nanostructured die with highly insulating square SiO_2_ spacers of 5 μm side length and 60 nm height was used as the counter electrode (Novocontrol, Germany). The geometrical and analytical details of the thin-film dielectric measurements with nanostructured counter electrodes are discussed in detail in our recent article.^45^

To confirm that the ultrastable CXB glasses can be indeed obtained by the PVD technique, we carried out two different sets of experiments in which the substrate temperature was maintained at either ∼277 K (0.85*T*_g_) or 333 K (1.02*T*_g_). The deposition rate was ∼0.22 nm/s, and the target film thickness was 400 nm in both cases. After preparation, the dielectric loss curves were recorded, demonstrating a higher onset temperature for 0.85*T*_g_ vapor-deposited CXB. In the second run, after the melting, this sample behaves just like an ordinary quenched-cooled liquid. In contrast, for the sample vapor-deposited at substrate temperature 1.02*T*_g_, no changes in the glass-transition dynamics were observed (please see Figure S4 in Supporting Information).

## Results and Discussion

### α-relaxation Dynamics and Crystallization of Vapor-deposited Thin Films

**Figure 1:**
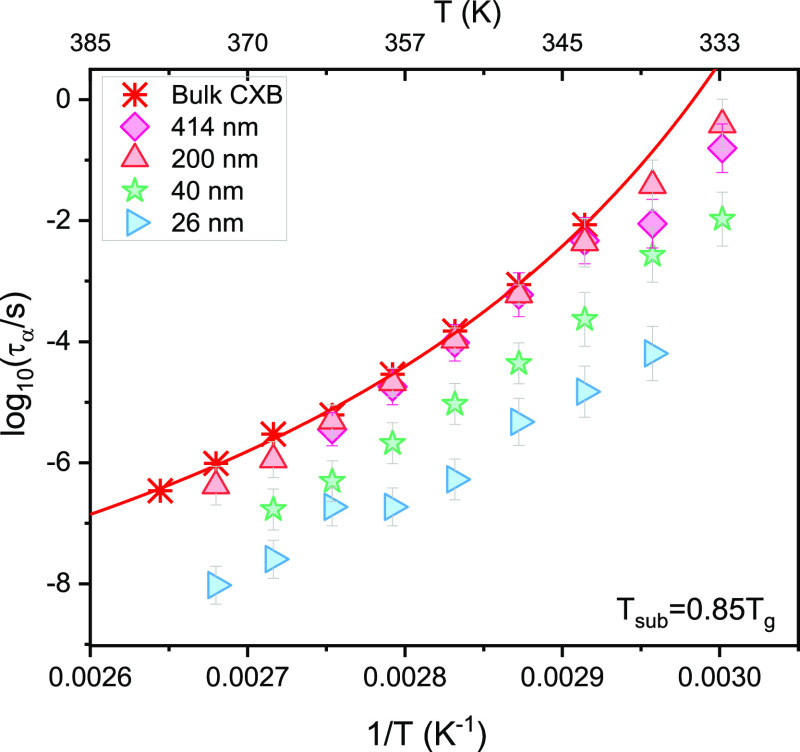
Temperature evolution of α-relaxation times for vapor-deposited CXB films with different thicknesses. The red line represents the VFT fit for bulk material.

One of the main reasons for the superior properties of vapor-deposited glass is its enhanced mobility at the surface.^21,46^ To understand the structural relaxation dynamics of vapor-deposited films, the α-relaxation times for bulk as well as vapor-deposited films of CXB with different thicknesses were extracted from the raw dielectric data (the modeling and analysis details are discussed in detail in our recent article^45^). Figure 1 shows the temperature dependence of α-relaxation time, τ_α_, for bulk as well as vapor-deposited films of CXB. The data were fitted using the Vogel–Fulcher–Tammann (VFT) equation^47,48^
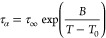
1where τ_∞_, *B*, and *T*_0_ are the fitting parameters that depend on the material.

The vapor-deposited films of CXB with thicknesses 414 and 200 nm follow bulk-like dynamics, whereas the mobility associated with the α-relaxation is faster when the film thickness reduces. The thinnest film thickness obtained in this work film, 26 nm, exhibits the fastest dynamics compared with other samples. This indicates a systematic enhancement of the α-relaxation dynamics of vapor-deposited CXB films with the reduction of film thickness. A reduced glass-transition temperature in thinner films was reported on vapor-deposited films by nano-calorimetry.^37^ It was also demonstrated that the position of the glass-transition peak shifts to higher temperatures as thickness increases and tends to stabilize at a certain thickness when the bulk transformation sets in.^49^ Besides, the tobacco mosaic virus-probe method applied when studying nanometric range thin films of organic molecular glass TPD has demonstrated that the surface diffusion coefficient is invariant of the film thickness and decouples from the glass-transition dynamics enhanced by 6–14 orders of magnitude.^50^ Based on that observation, it was concluded that the fast mobility at the surface might be a distinct process independent from the relaxation dynamics within the film. Nevertheless, a more recent observation indicates that vapor-deposited glasses can be made even without significant surface diffusion.^24^ There is also existing evidence that the highly mobile surface layer is only a few nanometers,^51^ which can probably be why its influence is more pronounced in the films of lower thickness. Similar behavior of faster dynamics in 1D confinement is also observed for polymer thin films as well as confined liquids.^52−54^

**Figure 2:**
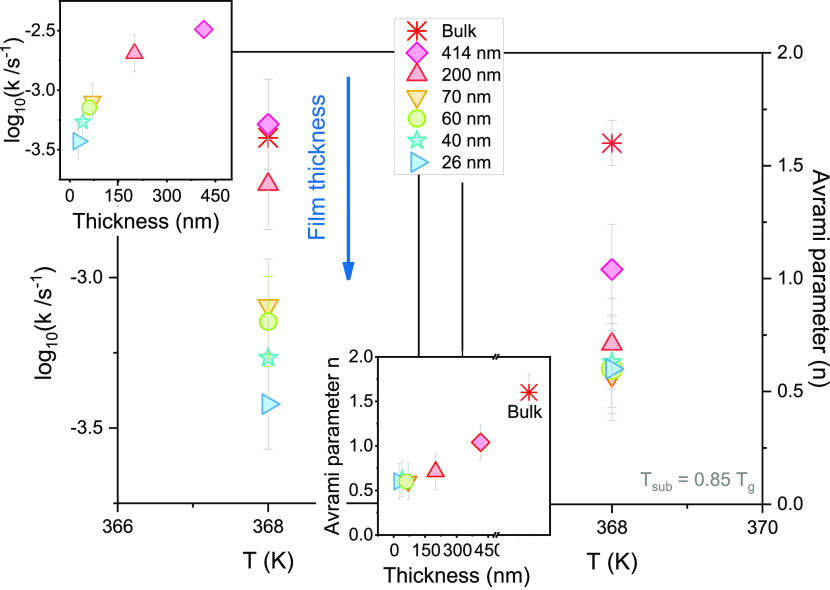
Crystallization rate *k* as a function of temperature (on the left) and the Avrami parameter (*n*) (on the right) for CXB films prepared using the vapor deposition technique as measured at 368 K for different thicknesses. In the insets, the crystallization rate and the Avrami parameter are presented as a function of the film thickness.

When it comes to crystallization, vapor deposition is reported to be a practical method that can resist the crystallization of organic glasses. Slowing down of the crystallization rate was observed for vapor-deposited organic molecules not only below *T*_g_(44,55) but also above it.^56^ Moreover, for the organic semiconductor CBP, the choice of the substrate deposition temperature can result in the procurement of two different polymorphic forms.^57^ For this reason, to understand whether the confinement further affects the crystallization kinetics of vapor-deposited films, we have conducted time-dependent studies under isothermal conditions. The changes in the real ε′ and imaginary ε″ parts of the dielectric permittivity were monitored above *T*_g_ at *T* = 368 K for every 10 s. The analysis of crystallization kinetics was conducted using the Avrami equation.^58^ In such a case, the transformed crystalline volume fraction can be described as
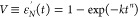
2where *k* is the rate constant, and *n* is the Avrami parameter. The crystallization rate provides combined information about the rate of nucleation (*N*) as well as the crystal growth (*G*), where *k* = *NG*^*n*–1^. Dielectric results and analysis of the crystallization kinetics data for vapor-deposited CXB are given in the Supporting Information (Figures S4 and S5). In Figure 2, we show the rate of crystallization which decreases with decreasing the film thickness of the vapor-deposited CXB films. Considering the Avrami parameter, irrespective of the film thickness, the value is between 0 and 1, which points to surface crystallization in one dimension.^59^ Also, the value of the Avrami constant obtained for the confined films appears to be less compared to the bulk. The theoretical predictions based on the isothermal crystallization of spherical entities in the limited volume suggest slower crystallization kinetics and a decrease in the Avrami exponent with decreasing thickness,^60^ which is well in agreement with the computer simulations as well as numerous experimental results discussing the crystallization of polymer thin films.^61−64^ The study of depth of penetration of surface crystals at the growth front suggests that at a steady-state, an advancing growth front does not have a substantial portion beneath the surface. If that portion is thinned down to ∼1 μm, a perturbed growth process should be expected.^65^ This can be very well related to our results as we see a decrease in the crystallization with decreasing film thickness. Hence, the slowing of the crystal growth can be attributed toward the lack of material as opposed to the actual kinetics of the crystal growth process.

After crystallization, crystallized films were heated to record the melting temperature. As it turned out, the melting temperature for 200 and 70 nm samples agreed with that reported for the bulk material. On the other hand, a decreased *T*_m_ value was observed for the thinnest vapor-deposited films of CXB, 26 nm (Figure S7 in Supporting Information). A shift in the melting temperature is one of the most characteristic confinement effects frequently reported for molecular systems in nanopores.^66^ Since the melting points of the three so far reported polymorphs of CXB are located close to each other (within ±2 K), we cannot exclude the fact that PVD samples might induce some polymorphic transformation. Nevertheless, one should also remember that the melting temperature shift observed with increasing confinement is often related to reducing crystal sizes.

**Figure 3:**
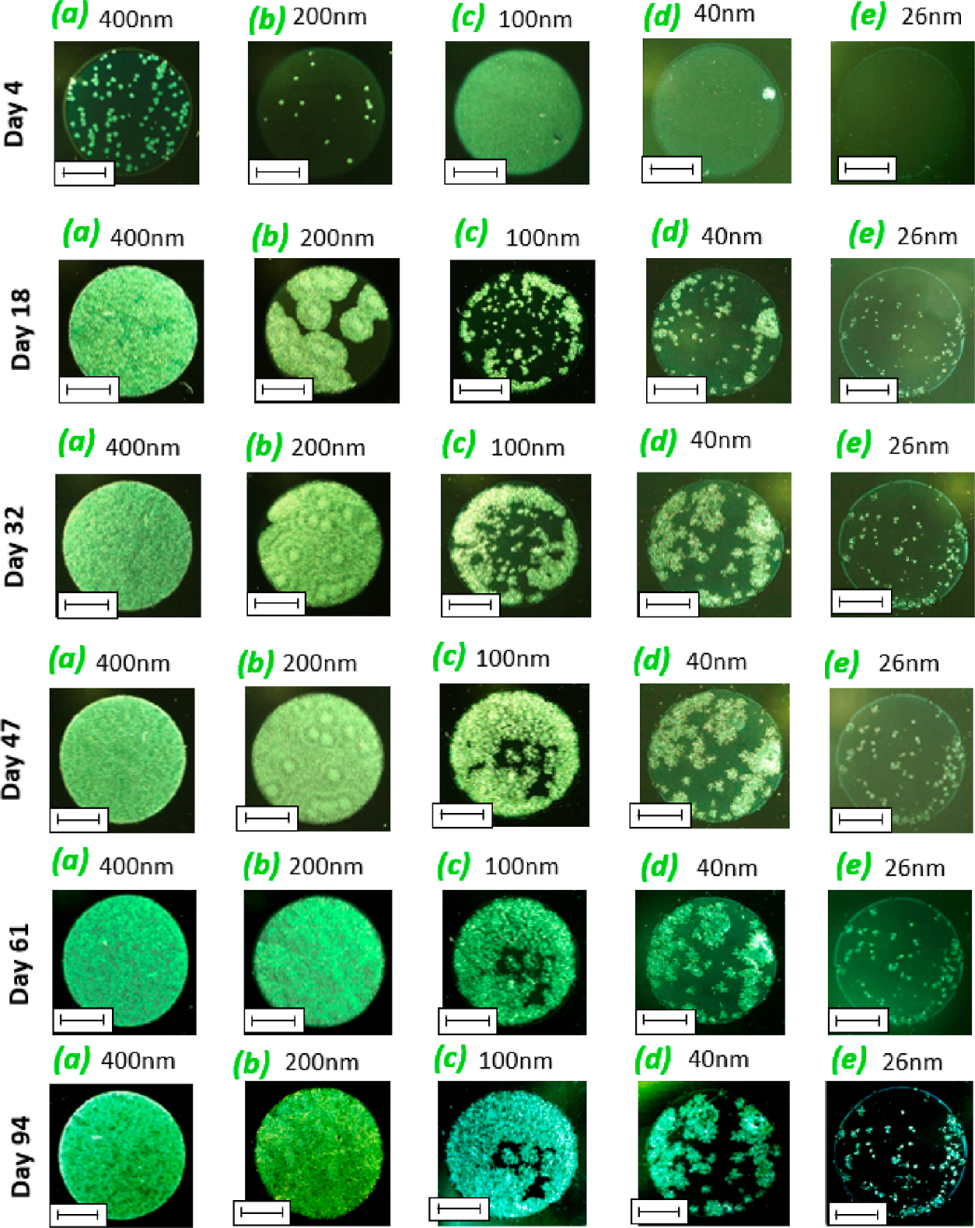
Optical microscopy images depicting the crystallization of vapor-deposited CXB films of various thicknesses on Si wafers with time as measured at room temperature. The scale bar represents ∼3 mm.

Apart from the crystallization kinetics study at 368 K, we have also followed the crystallization behavior of vapor-deposited CXB films by employing the optical microscope. In this case, experiments were carried out at room temperature, that is, in the glassy state. Figure 3 shows the crystallization of CXB films of various thicknesses with time. One can observe that thicker films crystallize faster compared to thinner films. Notably, the 26 nm film did not fully crystalize even after 94 days. The magnified version of Figure 3 showing details of the growing crystal structure is presented in the Supporting Information (Figure S8). Therein, we also provide enlarged images of the vapor-deposited films taken just after deposition and after some time. The as-prepared vapor-deposited films of CXB are uniform and continuous. However, with time, crystals appear and grow. The remaining liquid part (in between the crystalline spots) is still uniform. No dewetting was observed on the noncrystallized part of the film. Various studies have already reported that the rate of crystallization is considerably lower for vapor-deposited glasses.^44,56^ In addition, we found that the film thickness reduction can further slow down the crystallization rate. When the 400 nm film completely crystallized on the 18th day, the film of 26 nm crystallized only 23.8% of the total area. It is worth noting that even after 100 days, there is no notable progress in the crystallization of thinner films, especially at 26 nm.

Here, we would like to comment on different temperature and time scales of the dielectric and optical studies. The crystallization kinetics was inspected with the use of DS at 368 K, which is well above the glass-transition temperature. On the other hand, optical images were taken at room temperature, that is, below *T*_g_. The aim of using the optical method to follow changes in the crystallization behavior of CXB thin films was to cover the range of temperatures that cannot be studied in situ via DS. Therefore, in this way, we can obtain versatile information about the crystallization behavior of vapor-deposited films of CXB. Crystallization rates are extremely slow at room temperature and cannot be measured dielectrically by a standard setup. Below *T*_g_, we do not see α-relaxation in the dielectric loss spectra, so we cannot use it to track changes in the crystal fraction. Using secondary modes for that purpose might be questionable, especially since the modeling of the dielectric response of nanometric thin films in nanoelectrode arrangement configuration (to extract pure response of the confined sample) is based on Havriliak–Negami fits of well-pronounced α-relaxation.^45^ Moreover, below the glass-transition temperature, changes in the dielectric response of the sample due to crystallization might be overlaid with the aging process. For the reasons given above, determining the crystallization rates at room temperature via dielectric studies was not performed. Comparing the time scale of the crystallization process from optical and dielectric measurements above *T*_g_ might not be entirely accurate. This is because the crystallization rate determined from the dielectric studies incorporates combined information on both nucleation and crystal growth rates. Extracting their individual contributions is by no means possible. In contrast, the optical microscopy study provides information about the growth of the crystals but not the very early stages of the crystallization process.

### Comparison between CXB Deposited at a Substrate Temperature of 0.85*T*_g_ and That Deposited at Room Temperature

**Figure 4:**
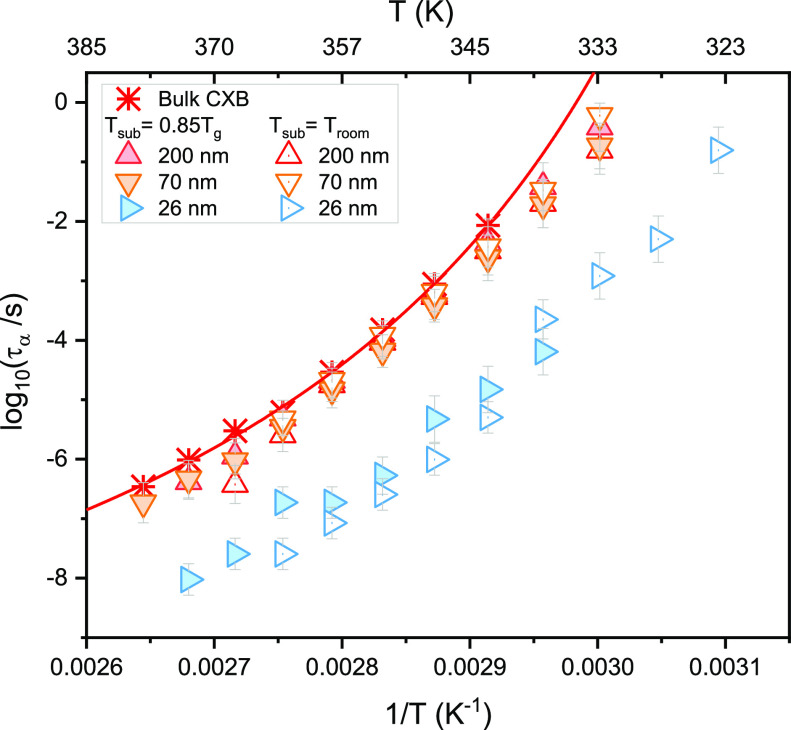
Temperature evolution of α-relaxation times for vapor-deposited CXB films with different thicknesses obtained while maintaining different substrate temperatures. The red line represents the best VFT fit for bulk material. *T*_room_ means the room temperature of the substrate for deposition, which is ∼0.9*T*_g_.

As highlighted in the introduction, it is widely reported that the deposition rate, as well as the temperature of the substrate during the deposition, substantially influence the properties of the vapor-deposited film, such as the enthalpy, stability, density, and so forth.^11^ Besides, recent studies have also shown that the temperature of deposition can also influence molecular packing.^43^ The optimum substrate temperature reported for the deposition is 0.85*T*_g_.^44^ Hence, our next aim is to compare the vapor-deposited CXB, deposited at a substrate temperature of 0.85*T*_g_ with that deposited at room temperature at various thicknesses. Figure 4 compares the temperature dependence of α-relaxation time, τ_α_, for PVD films of the same thickness but deposited at different *T*_sub_. Irrespective of the substrate temperature for vapor-deposited CXB films, τ_α_ follows almost the same temperature dependence.

**Figure 5:**
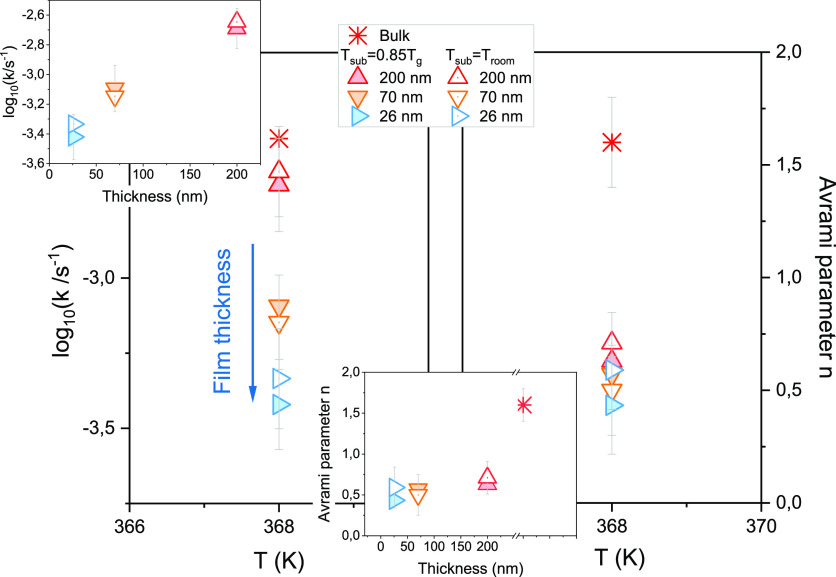
Crystallization rate *k* (on the left) and the Avrami parameter (on the right) for CXB vapor-deposited films with different thicknesses and substrate temperatures during deposition as measured at 368 K. *T*_room_ means the room temperature of the substrate upon deposition, which is ∼0.9*T*_g_. The inset plot thickness depends *k* and *n*.

Considering the crystallization behavior and the Avrami parameter, Figure 5 shows that the crystallization rate at 368 K is comparable for *T*_sub_ = 0.85*T*_g_ and *T*_sub_ = 0.9*T*_g_. The Avrami constant is also found to be independent of the substrate temperature for the vapor-deposited films. This suggests that the confinement by film thickness does not influence to hold back the properties due to the deposition temperature when the material is above its glass-transition temperature.

### Influence of the Thin-film Preparation Technique on the α-relaxation Dynamics and Crystallization Behavior of Confined CXB Films

**Figure 6:**
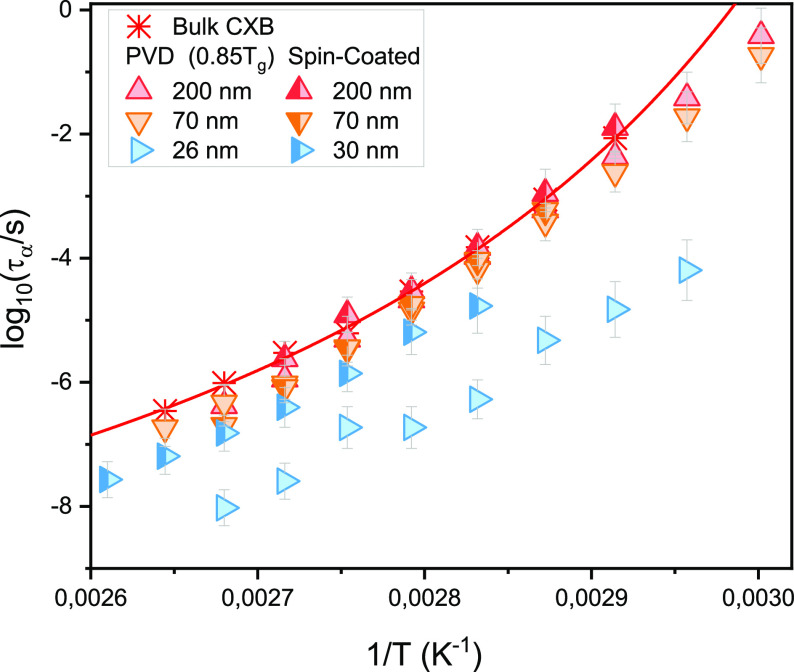
Temperature evolution of α-relaxation times for vapor-deposited and SC CXB films. The red line represents the best VFT fit for the bulk sample.

If two systems are in metastable equilibrium, their dynamics and crystallization should be the same. However, thin-film dynamics show numerous out-of-equilibrium features, where the preparation conditions, processing time scale, or thermal treatment protocols determine its behavior. The equilibration kinetics for such systems can exceed over time scales much longer than structural relaxation, so it freezes into a kind of nonequilibrium conformation. Therefore, thin films obtained via different processing pathways are known to exhibit dynamics varying locally in space and time.^67^ Nanometric confinement is known to induce spectacular changes in material properties and so do the film preparation techniques. Hence, it is interesting to decouple the effects caused by the thickness confinement that arose from different thin-film preparation techniques. In the following, we have systematically investigated molecular mobility related to the α-relaxation and crystallization behavior of vapor-deposited films with SC counterparts possessing similar thicknesses. Figure 6 shows a comparison of the temperature evolution of the α-relaxation times for vapor-deposited and SC films of the same thicknesses. Even though at 100 and 70 nm thicknesses, a difference in τ_α_(*T*) is not clearly distinguishable from the figure, a close examination shows that the α-relaxation dynamics is faster for the vapor-deposited films compared to their SC counterparts.

Nevertheless, one can clearly observe an enhancement of molecular mobility for vapor-deposited films at ∼30 nm. At this point, we would like to mention that the films after spin coating were annealed at 40 °C in vacuum for 3 h to facilitate residual solvent evaporation. In the presence of a residual solvent, the dynamics of SC films are supposed to be faster. Here, we observe faster dynamics for vapor-deposited films where no solvent was used. Intuitively, we do not expect differences in the structural dynamics between SC and vapor-deposited materials of the same thickness when warmed up above the glass-transition temperature. The results of this study demonstrate that this might not be essentially true because vapor deposition and SC films are not the same in many points. The early evidence of that comes from the literature findings. For example, it has been demonstrated for columnar liquid crystal OLEDs that the choice of the preparation method, SC versus vapor deposition, influences the molecular packing and the intermolecular order. A higher molecular order and, in consequence, improved charge carrier mobility were observed for vapor-deposited samples.^68^ SC organic molecules exhibit an isotropic glass structure, just like that expected for ordinary quenched-cooled liquids. On the other hand, PVD allows us to induce structural anisotropism.^35^ This occurs especially when the substrate temperature is kept at ∼0.85*T*_g_ during the vapor deposition; the anisotropic molecules can tend to orient parallel to the surface.^69^ Small-molecule films prepared by these two different routes might differ not only in molecular orientation but also in thermal stability and density. By studying changes in the thermal expansion coefficient for ∼70 nm films of OLED materials, Shibata et al. have demonstrated that the glass-transition temperature for vapor-deposited films occurs ∼10–20° above that found for bulk and SC films. Interestingly, in the first heating run, the evolution of the thermal expansion coefficient for the vapor-deposited film recorded well above *T*_g_ completely does not resemble that of the bulk and SC films, while it recovers the same temperature characteristics in the second heating cycle.^70^ Faster dynamics that we have observed for the vapor-deposited films can be well correlated with the observations by Franz et al. that low-molecular-weight molecules with a definite orientation to the substrate can stimulate faster dynamics.^71^

For low-molecular simple organic molecules, 2-methyltetrahydrofuran (MTHF) annealing of as-deposited films above *T*_g_ also results in obtaining unusual liquid states with reduced dielectric strength compared to the ordinary counterpart. As concluded from that study, when heated above *T*_g_, vapor-deposited MTHF does not transform into the same supercooled liquid state as that reached by cooling the ordinary melt. Therefore, it has been speculated that vapor deposition might promote polyamorphism and result in seeing distinct long-lived metastable states with different dynamics.^72,73^ CXB also possesses hydrogen bonding possibilities (two oxygen atoms of the sulfonamide group, fluorine atoms of the CF3 group, and two nitrogen atoms in the heterocyclic ring). Variable hydrogen-bond formation leads to an intermolecular association within CXB molecules which includes five-membered structures. FTIR studies have demonstrated a strengthening of hydrogen bonds in the amorphous state of CXB and related its thermodynamic behavior and physical stability behavior to interaction patterns at the molecular level.^74^ Peculiar behavior was also observed for vapor-deposited toluene films of ∼100 nm thickness, which upon annealing above *T*_g_ for a different amount of time, produce distinct glassy states. Nevertheless, the as-deposited toluene eventually transforms into ordinary glass when annealed for a sufficiently long time at a higher temperature. The time needed to transform the vapor-deposited sample into a supercooled liquid was found to be much longer than the α-relaxation time or as typically seen for ordinary glasses.^75^ Besides, the in situ observation of vapor-deposited organic glass also revealed the presence of a fast surface layer during PVD.^76^ Upon PVD, the molecules at the surface are highly mobile. This aids in sampling different ways of packing to attain a better glass structure/configuration before being overlayed by other deposited layers. A similar mechanism is probably not available upon rapid removal of the solvent by SC. Therefore, better packing of vapor-deposited films can possibly delay crystallization, while for SC samples, the structure is more loose, which should favor crystallization.

**Figure 7:**
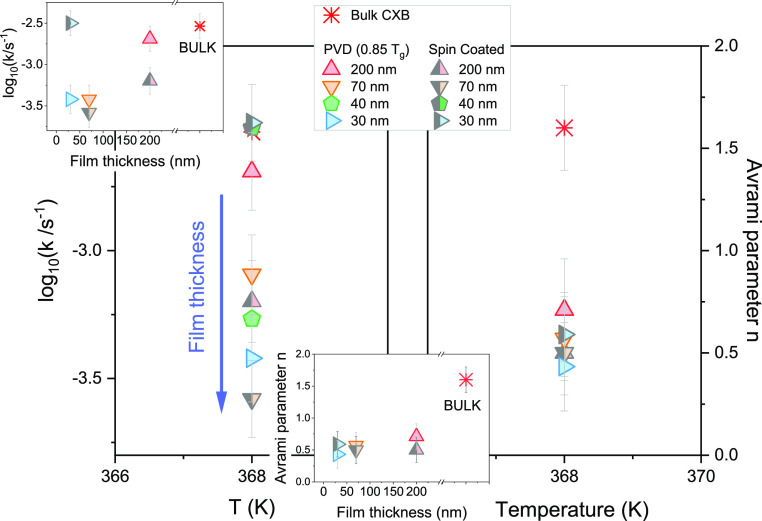
Crystallization rate constant (*k*) characterizing the rate of the crystallization process, on the left, and Avrami parameter (*n*), on the right, at 368 K, for CXB films of the same thicknesses but prepared using different methods. The inset plot thickness depends on *k* and *n*.

In Figure 7, we demonstrate the evolution of the crystallization rate and Avrami parameter for CXB films prepared by different routes and investigated at 368 K after heating from the glassy state. Comparing the isothermal crystallization results, we have observed that 200 and 70 nm vapor-deposited CXB films crystalize faster compared to the SC films of the same thickness. On the other hand, by looking at the changes in the Avrami parameter, we see a trend for lowering the n value with decreasing film thickness but no pronounced differences between SC and vapor-deposited films.

The faster crystallization observed in the vapor-deposited films can be attributed to their high molecular orientation compared to the SC films, especially as they are deposited at a controlled substrate temperature.^35,70^ The molecular dynamics simulations showed that the bond-orientational order could lead to a faster crystallization process, where the vapor-deposited films are known to generate highly ordered films compared to SC films.^77^ There is an exception to the general trend when considering 40 and 30 nm SC films. They follow a bulk-like crystallization rate, whereas the vapor-deposited films follow the general trend, that is, slower crystallization with decreasing film thickness. In this case, one should consider the very high density of CXB at the substrate and the increased free surface effect in vapor-deposited films. In contrast, the bulk-like behavior of SC films of similar thickness has been previously observed in polymer thin films, where both the substrate and the free surface can significantly influence the ultrathin film properties.^54^

## Summary

This study puts forward the understanding of glass-transition dynamics and crystallization behavior of vapor-deposited glasses under geometric 1D confinement. We found that the vapor-deposited films exhibit faster dynamics as the film thickness decreases. It is also evident that the crystallization tendency slows down with the increasing confinement (reduced film thickness) of vapor-deposited CXB films, both above and below the glass-transition temperature. Even when heated above *T*_g_, the properties of vapor-deposited films do not cease nor transform back into a regular liquid. However, we found marked differences between glass-transition dynamics and crystallization tendency of vapor-deposited films and SC films of the same thicknesses. The fact that the material can remain amorphous for a prolonged period of time when it is confined by the thickness is promising for a wide range of technological as well as pharmaceutical applications. Thus, the PVD together with the 1D thickness confinement can bring about a promising way to keep the amorphous glasses stable for a prolonged period of time.

## Supporting Information Available

Differential scanning calorimetry (DSC) measurements of bulk CXB; details of physical vapor deposition system; dielectric loss data for vapor-deposited celecoxib (CXB); isothermal crystallization of vapor-deposited CXB films studied by dielectric spectroscopy; melting behavior of vapor-deposited CLX films; optical microscopy images of CXB crystallization with time; and additional snapshots/optical microscope images of CXB films taken just after deposition and after six months of storage (PDF)
