# Supplementary material for: Vapor-Deposited Thin Films: Studying Crystallization and α-relaxation Dynamics of the Molecular Drug Celecoxib
Source: J Phys Chem B. 2022 May 17;126(20):3789–98. doi: 10.1021/acs.jpcb.2c01284 (PMC9150116; doi:10.1021/acs.jpcb.2c01284)
Supplement: Supplementary file 1 — jp2c01284_si_001.pdf [file jp2c01284_si_001.pdf]

Supporting Information for

Vapor deposited thin films: Studying crystallization and α-relaxation dynamics of molecular drug celecoxib

Aparna Beena Unni,[†,‡]* *Roksana Winkler,*[†,‡] *Daniel Marques Duarte,*[†,‡] *Wenkang Tu,*[†,‡]

Katarzyna Chat[†,‡] *and Karolina Adrjanowicz*[†,‡]*

[†] Institute of Physics, University of Silesia, 75 Pulku Piechoty 1, 41-500 Chorzow, Poland

[‡] Silesian Center for Education and Interdisciplinary Research (SMCEBI), 75 Pulku Piechoty 1a, 41-500 Chorzow, Poland

Corresponding Authors

* aparna.beena-unni@smcebi.edu.pl, kadrjano@us.edu.pl

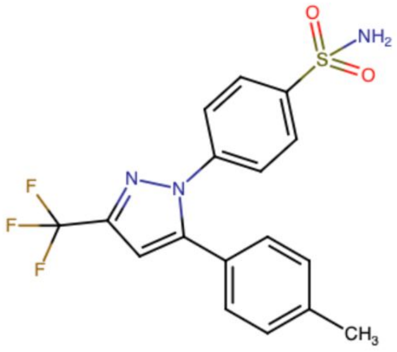

Figure S1. Molecular structure of celecoxib, CXB, compound investigated in this study.

Differential Scanning Calorimetry (DSC) measurements of bulk CXB

Thermodynamic properties of the tested material were investigated using a Mettler-Toledo DSC apparatus equipped with a liquid nitrogen cooling accessory and an HSS8 ceramic sensor (a heat flux sensor with 120 thermocouples). Indium and zinc standards were used for the temperature and enthalpy calibrations. The tested sample was placed in aluminum crucibles and sealed. Experiments were performed for the bulk CXB (~ 10 mg) within the temperature range of 273 K - 453 K. The heating and cooling runs were carried out at the rate of 10 K/min. The crystalline sample was first kept at T= 353 K for one hour under nitrogen flow to remove water absorbed upon sample preparation. Subsequently, CXB was heated up to T= 453 K, i.e., above the melting point. The melted sample was then cooled to T= 273 K to vitrify. As a next step, the glassy form of CXB was reheated to T= 453 K. From the DSC heating thermograms, characteristic temperatures, including the melting point T_m, glass transition temperature T_g, and cold crystallization temperature T_c are determined. The melting point of the investigated compound is 435 K, while its glass-transition temperature T_g=326.6K. As demonstrated in Figure S2, upon heating from the glassy state, recrystallization of celecoxib takes place at ~390 K. Knowledge of T_g, T_c, and T_m values were necessary for setting the evaporation conditions as well as selecting the optimal temperature range for crystallization studies.

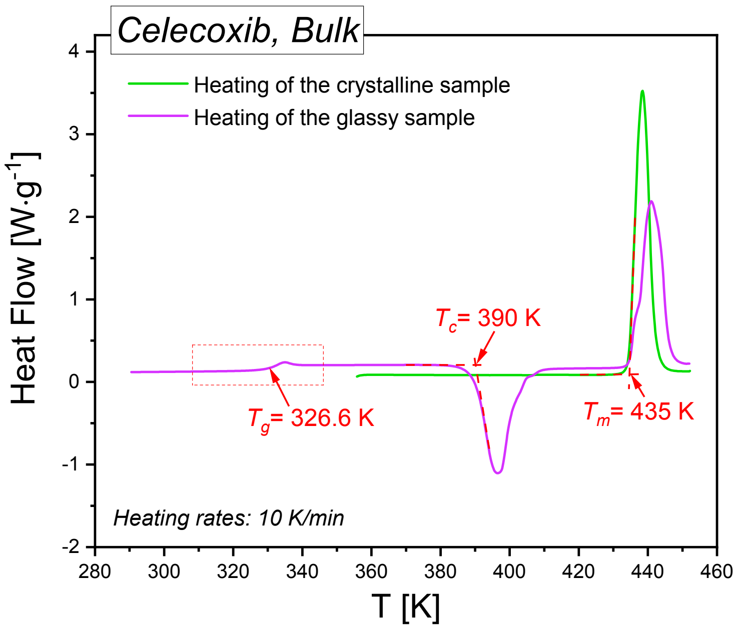

Figure S2. DSC thermograms recorded upon heating crystalline and glassy forms of celecoxib with the rate of 10 K/min. The glassy sample was prepared by quenching the melt with 10 K/min. Thermal events associated with the melting, glass transition, and cold crystallization phenomena are shown. T_g is calculated as the midpoint between onset and endset of the step changes in the heat flow. Cold crystallization temperature, T_c , and melting temperature, T_m, are determined from the onset temperatures of the corresponding processes.

Physical Vapor Deposition System

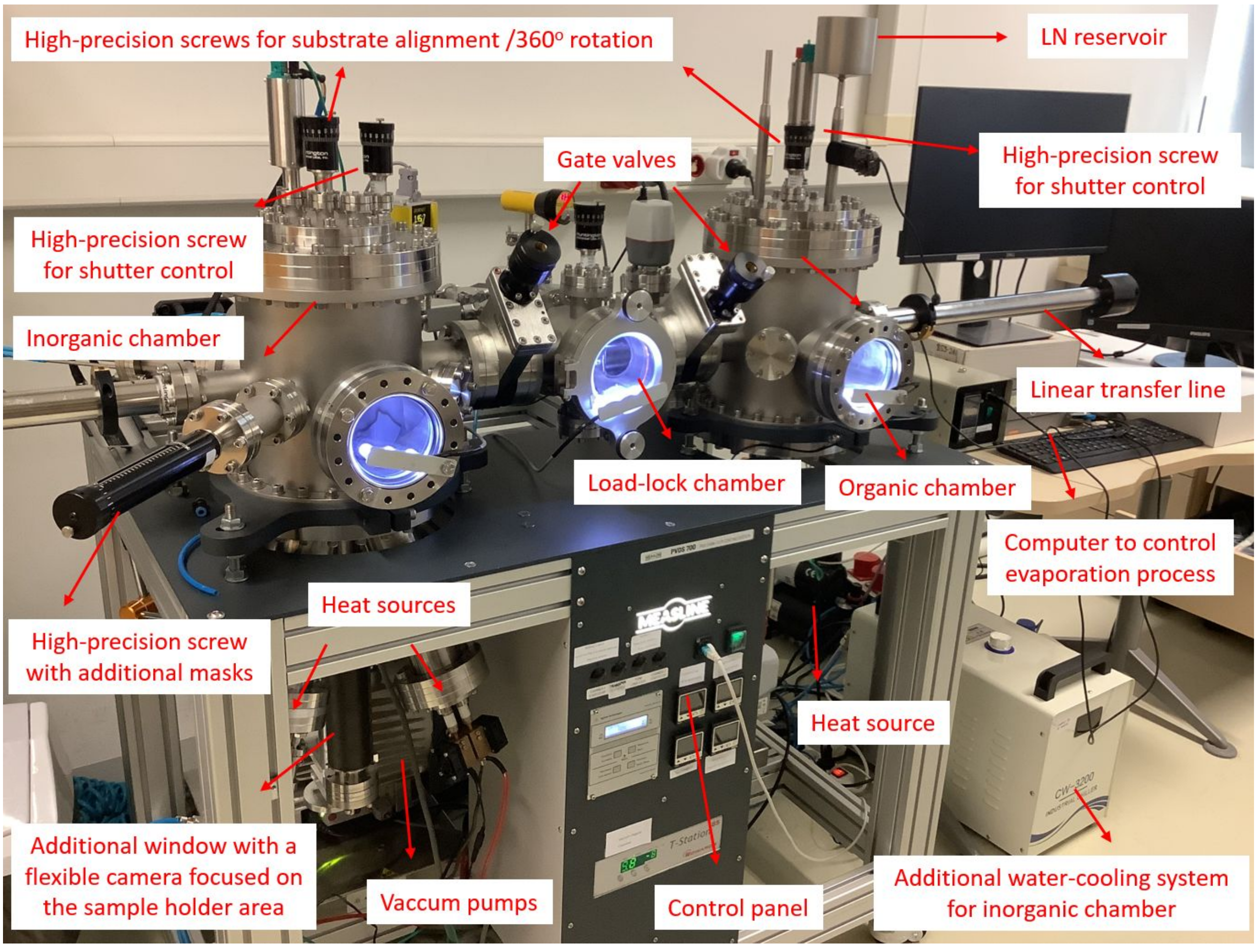

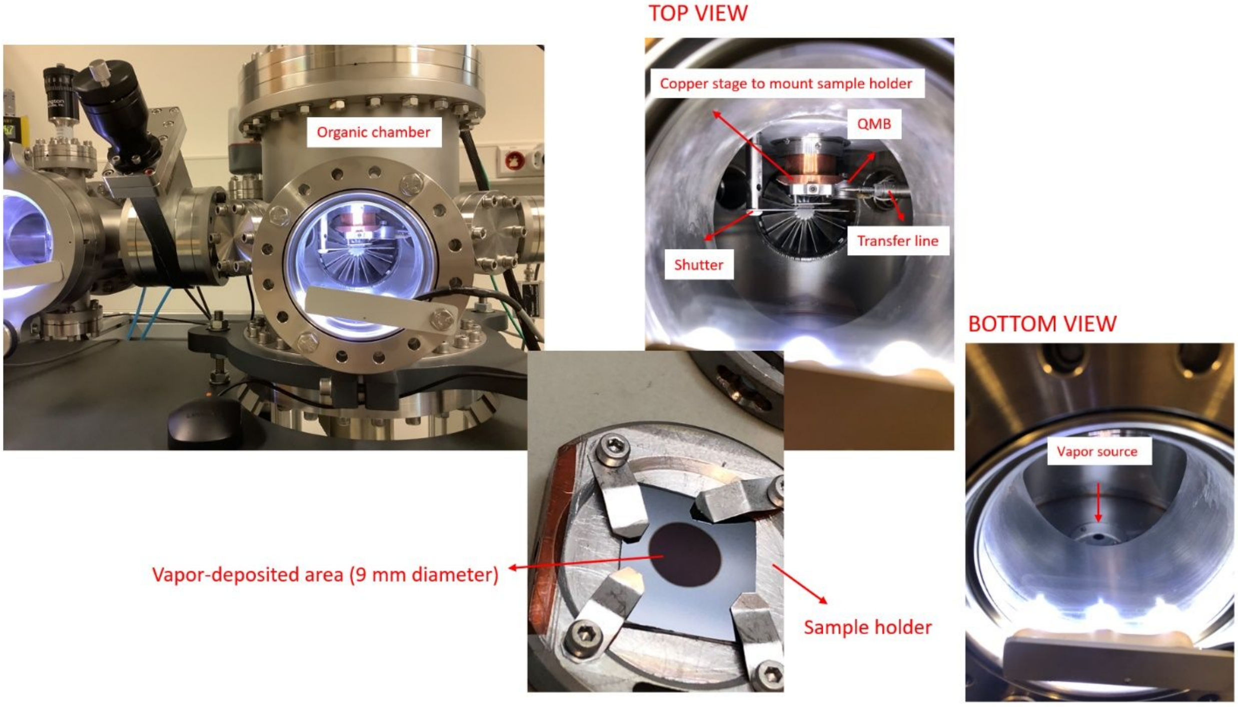

Figure S3. Schematic view of PVD system used in this study.

High-vacuum conditions essential for thermal evaporation were achieved using Edwards nXDS10i dry scroll pump for initial pumping down to mbar range, and continued by the turbomolecular station (Edwards T-Station 85H DRY CF63) until ~$2{\cdot}10^{-8}$ mbar. The vacuum pressure was measured by Edwards's gauge WRG DN40CF. Our system performs thermal evaporation of the materials. It is possible to evaporate materials with a base pressure of 10^{-8} mbar. Samples (wafers, plates, substrates) are mounted on copper holders and fixed using 4 S-Clips. The temperature of the copper sample holder is controlled by external thermoelectric temperature control systems. The substrate can be cooled down below room temperature using a liquid nitrogen reservoir. The temperature is controlled with a precision better than 0.1 K. The temperature on the sample holder mounted inside the chamber was also tested using an external thermocouple. Upon deposition, it is possible to rotate the substrate holder at a constant rate using an external motor to obtain better homogeneity of the films. The materials to be evaporated are loaded into crucibles made of alumina. The crucibles mounted on the filaments are heated using an integrated power source, which allows controlling the temperature with 0.1 K precision. Then, the materials are thermally evaporated. As the temperature increases, the rate of deposition increases. It can be controlled manually by adjusting the temperature/input power. The rate of deposition and the thickness of the deposited layers in both chambers are monitored using quartz microbalances (EDF Electronics QM20). In the standard position, the shutter placed approximately 10 cm away from the sample holder protects the substrate area so that the evaporated species do not hit the surface area at any

time. Only when the deposition rate is stable, the shutter is opened, allowing the material deposition on the substrate. Evaporation is carried out until desired thickness of the sample is obtained. Then, the shutter is closed again, and the temperature power source is switched off. Whenever needed, the evaporation area can be maintained by using circular masks of varying diameters while the rest of the surface remains protected from being coated. The deposition is typically carried out within a circular area of diameter ~ 9 mm. As a final step, the substrate temperature is either increased/decreased to go back to room temperature.

Dielectric loss data for vapor-deposited celecoxib (CXB)

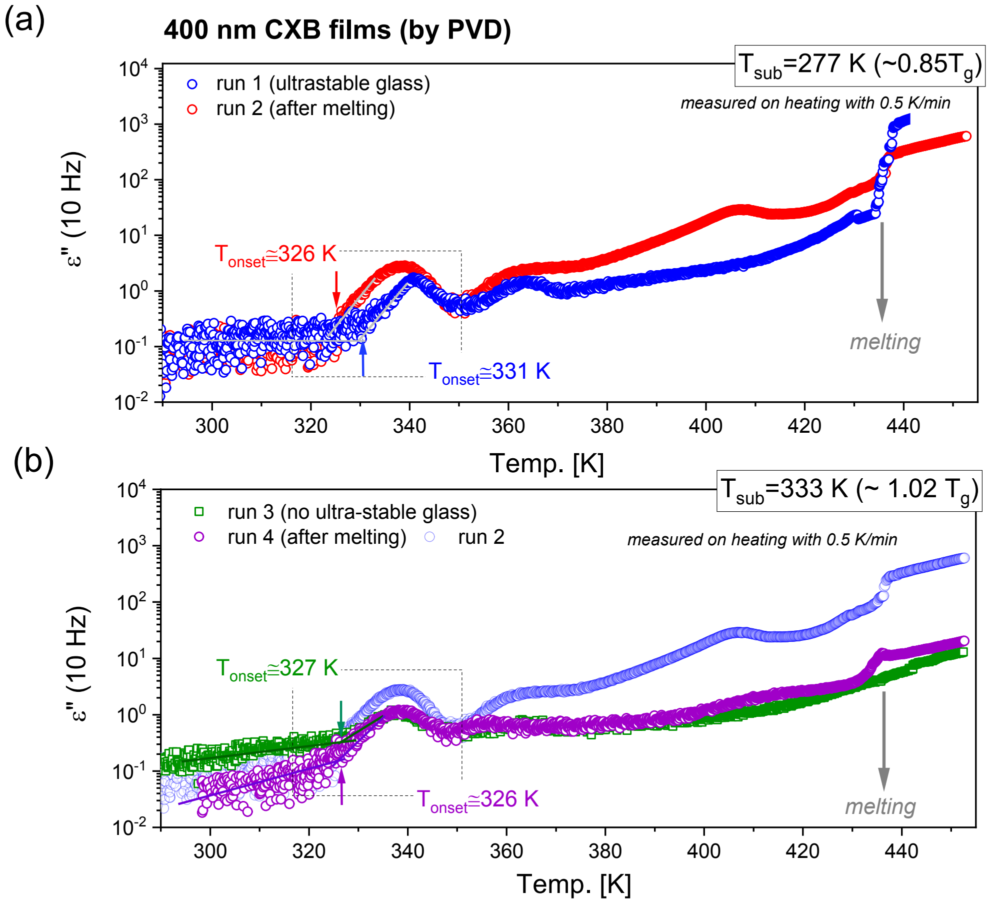

Figure S4. Dielectric permittivity ε" at a test frequency *f*=10 Hz measured on heating with 0.5 K/min for celecoxib deposited on silicon wafers with two different substrate temperatures: T_{sub}=277K (panel a) and T_{sub}=333K (panel b). The deposition rate is ~ 0.22 nm/s, and the film thickness 400 nm.

To confirm that the ultra-stable glass of CXB was indeed produced by the PVD technique, we have performed temperature-ramping experiments following a similar protocol and dielectric data interpretation as can be found in the literature. [1–3] Here, the dielectric response was monitored for two samples of the same thickness (~400 nm), prepared with the same deposition rate (~0.22 nm/s) but different substrate temperatures ($0.85T_g$ and $1.02T_g$). Figure S4 shows changes in the dielectric loss curves at 10 Hz as measured upon heating of vapor-deposited celecoxib with the target thickness of 400 nm. Additional heating scans (run 2 and run 4) were performed on vapor-deposited CXB after completing melting in runs 1 and 3, respectively. The results are presented in Figure S4, panels a and b.

To understand the ongoing changes, we start with the sample deposited at $0.85T_g$ and the first heating scan (run 1). The dashed region in Figure S4a shows a temperature region of interest. A characteristic peak seen in dielectric loss spectra most probably does not represent the proper α-relaxation. Still, it is distorted due to the conversion of the ultra-stable glass to the ordinary supercooled liquid. Heating the sample much above the glass-transition temperature erases the history, and subsequent cooling produces only an ordinary glass. When compared with the results taken after complete melting of the sample in run 2, we found that the onset temperature seen in the first scan is shifted towards higher temperatures. The onset temperature is the temperature at which the glass begins to transform into the supercooled liquid. A shift of the onset temperature signifies increased kinetic stability of vapor-deposited at $0.85T_g$ CXB compared to the ordinary glass formed upon cooling the melt.

In contrast, the dielectric response at 10 Hz for CXB deposited at $1.02T_g$ shows no significant differences in the considered temperature region for as-deposited and melt-cooled samples (runs 3 and 4, Figure S4b). In this case, the onset temperature is ~326 K and indicates that the glassy dynamics of CXB deposited above T_g is essentially the same as for the ordinary supercooled liquid.

The second interesting feature revealed in this experiment is the reduced loss values for ultra-stable CXB glass prior to its transformation into supercooled liquid (run 1 vs. run 2). It has been suggested in the literature that such effect could indicate suppression of the secondary relaxation as measured

at a fixed frequency as a function of time. [1,2] After conversion, within the temperature range ~345-350K, the values of ε" recorded in runs 1 and 2 coincide. However, with increasing the temperature, they again start to diverge. Here, we want to mention that celecoxib is prone to recrystallization when heated from the glassy state. It is also known that the crystallization tendency of vapor-deposited and ordinary CXB glass is much different. Therefore, the dielectric loss spectra observed in the temperature range from 345K to 435K are most probably affected by partial recrystallization of CXB. The melting event seen as a sudden increase of ε" values at ~436 K was indeed detected in each heating run (1-4), meaning that the re-crystallization process takes place on increasing the temperature.

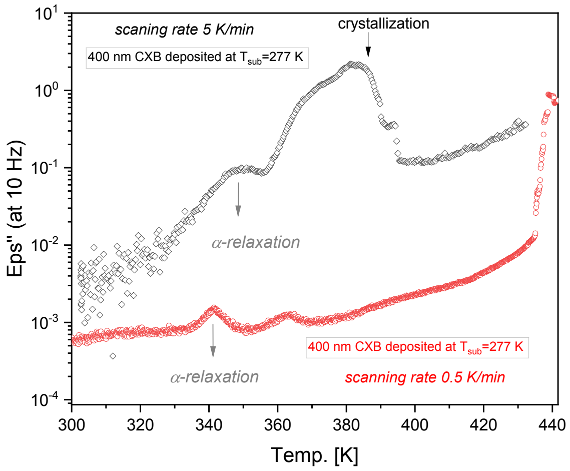

Figure. S5. Dielectric permittivity ε" at a test frequency f=10 Hz measured using two different scanning rates: 5 K/min and 0.5 K/min for celecoxib deposited on silicon wafers with the substrate temperature of 277 K. The deposition rate is ~ 0.22 nm/s, and the film thickness 400 nm.

Isothermal crystallization of vapor-deposited CXB films studied by dielectric spectroscopy

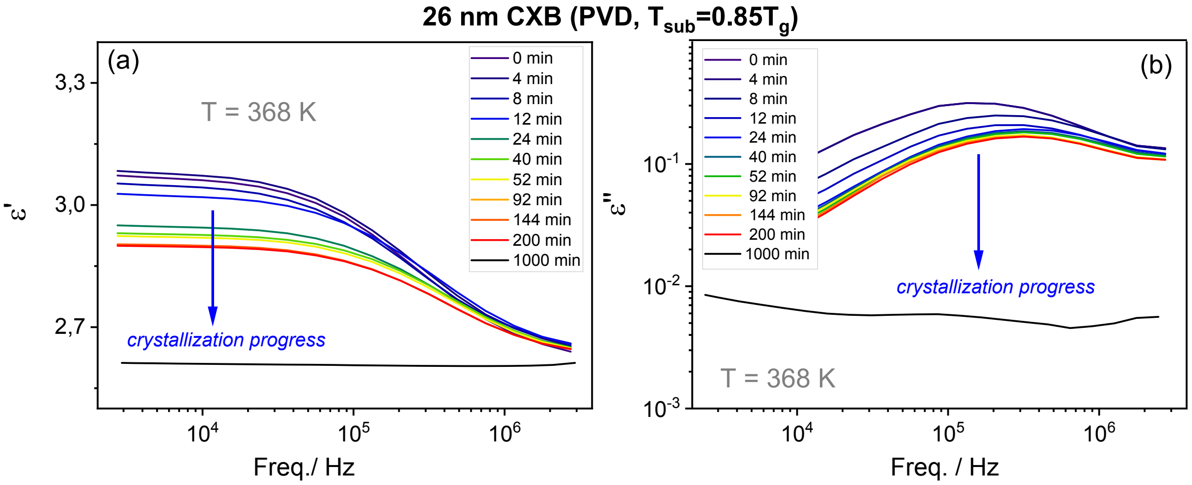

Figure S6. Changes in the (a) real and (b) imaginary parts of the complex dielectric permittivity (b) during the isothermal crystallization of 26 nm vapor-deposited CXB film (T_{sub}=0.85T_g) as monitored at 368 K.

Crystallization progress can be followed by using dielectric spectroscopy. Figure S6 shows the evolution of the real and imaginary parts of the complex dielectric permittivity as measured at 368 K for 26 nm vapor-deposited CXB film. An observed drop of the strength of the α-relaxation upon time-dependent measurements signifies crystallization progress. The crystallization kinetics was analyzed by following changes in the dielectric permittivity for the selected frequency (static limit), which was normalized based on the following formula

$$\varepsilon'_N(t) = \frac{\varepsilon'(t=0) - \varepsilon'(t)}{\varepsilon'(t=0) - \varepsilon'(t=\infty)} \quad \text{(S1)}$$

where ε′(t) is the value of the static dielectric permittivity for the selected frequency at a given time t, ε′(t = 0) is the value of the static dielectric permittivity for the same frequency chosen at the beginning of the crystallization, and ε′(t = ∞) is the long-time limiting value. The representative crystallization kinetic curves obtained for CXB vapor-deposited films with different thicknesses are shown in Figure S7. We find crystallization rate and Avrami parameter by fitting the data using the Avrami equation (Eq. 2 in the main paper). The results presented in Figure S7 and Figure 2 (from the main text) make evident that the crystallization slows down with decreasing thickness of vapor-deposited CXB films.

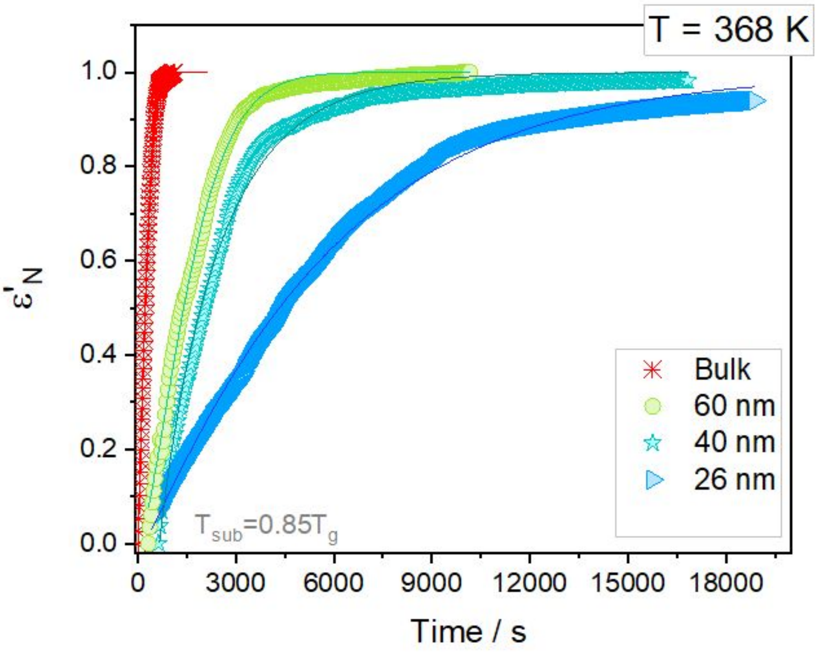

Figure S7. Isothermal crystallization of CXB at 368 K demonstrates the evolution of normalized dielectric permittivity with time for bulk and vapor-deposited CXB of different film thicknesses. Solid lines are Avrami fits to the data.

Melting behavior of vapor-deposited CLX films

To detect the melting point for nanometric size vapor-deposited CXB films temperature-ramp dielectric measurements were performed. After completing the isothermal crystallization experiment at 368 K, a temperature was increased with a ramp of 0.5 K/min, and complex dielectric permittivity at 10 kHz was recorded continuously until it reached 440-450 K. In Figure S8, we show results for three different CXB films with various thickness: 26 nm, 70 nm, and 200 nm. A sharp increase of the dielectric permittivity signifies a melting event (upon transformation to a liquid state, the mobility of the relaxing dipolar molecules is freed, while in the crystalline form, they do not contribute to the total dielectric response of the sample). As can be seen, the value of T_m for thicker films agrees very well with that reported for bulk CXB. On the other hand, for 26 nm film, the onset of the melting point is shifted slightly to lower temperatures. As noted in the main paper, a shift in the melting temperature is one of the most characteristic confinement effects and can be ascribed to the limitation in the crystal size.

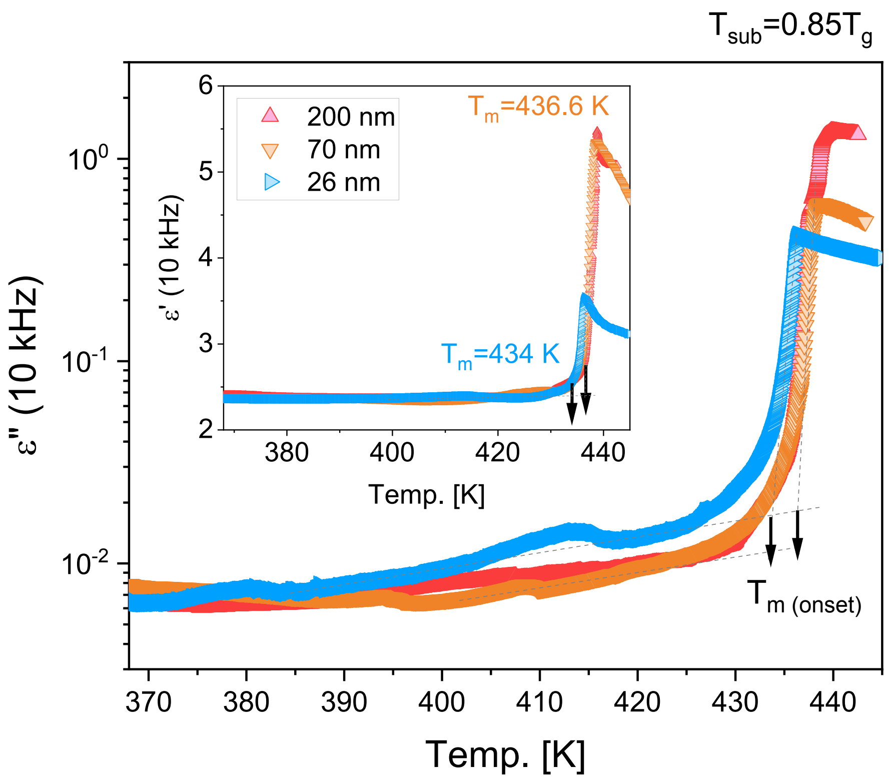

Figure S8. Temperature dependence of the complex dielectric permittivity: imaginary part ε'' (main figure) and real part ε' (inset) recorded at 10 kHz upon heating of crystallized vapor-deposited CLX films of various thicknesses. The heating rate is 0.5 K/min. The melting point was determined from the onset temperature of the transformation.

Optical microscope images of CXB crystallization with time

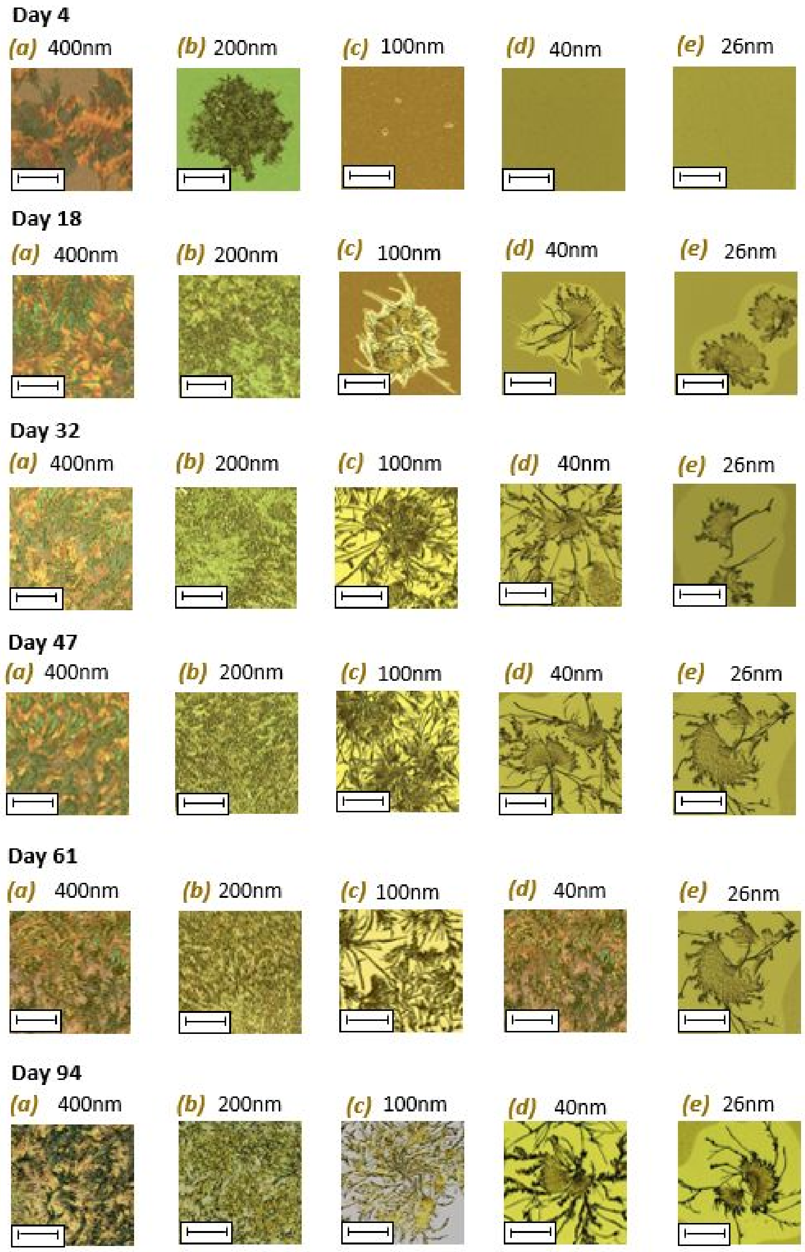

Figure S9. Zoomed optical microscope images depicting the crystallization of vapor-deposited CXB films of various thicknesses on Si wafers with time. The scale bar represents ~0.1 mm. The measurements were carried out at room temperature.

Additional snapshots/optical microscope images of CXB films taken just after deposition and after six months of storage.

PVD of CXB was done in a circular region with ~ 9 mm diameter using silicon wafers with a native oxide layer as a substrate. As-prepared vapor-deposited films of CXB are uniform and continuous. However, with time crystals appears and grow. The remaining liquid part (in between the crystalline spots) is still uniform. No dewetting was observed. Optical microscope images cover the size 330 microns x 250 microns.

Just as prepared vapor-deposited CXB films of various thickness

Figure. S10. (a) 26 nm deposited film

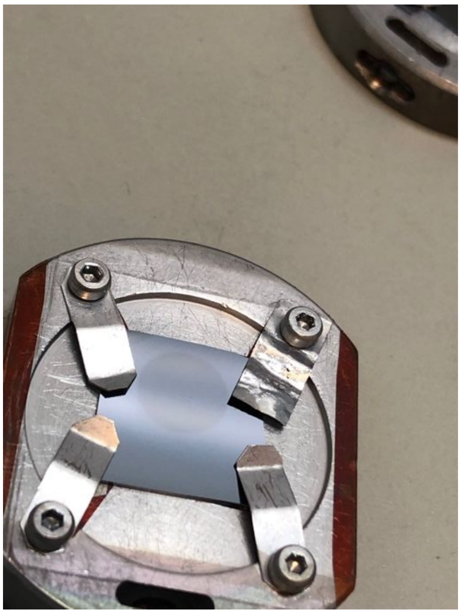

Optical microscope image of 26 nm sample position 1

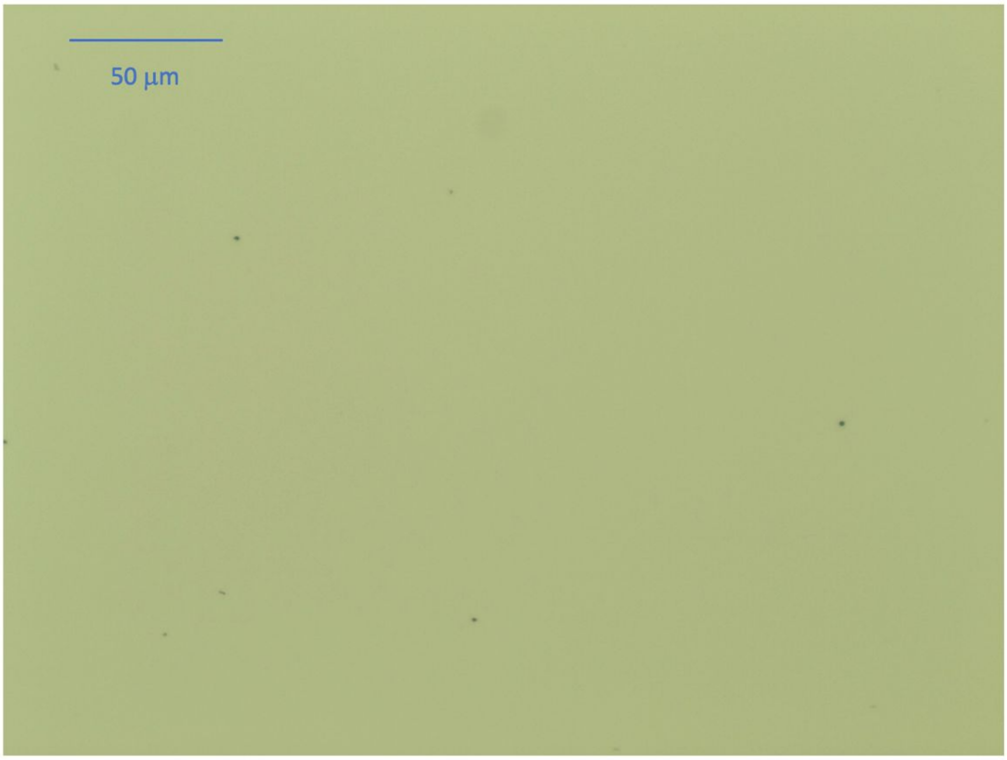

Optical microscope image of 26 nm sample position 2

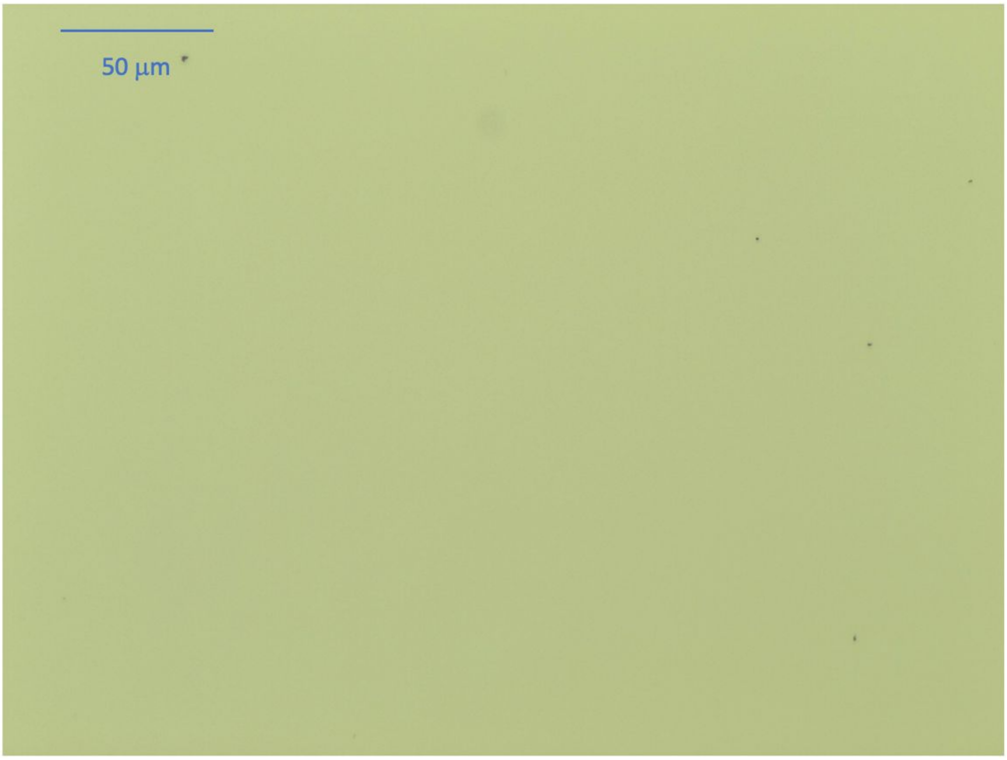

Optical microscope image of 26 nm sample position 3

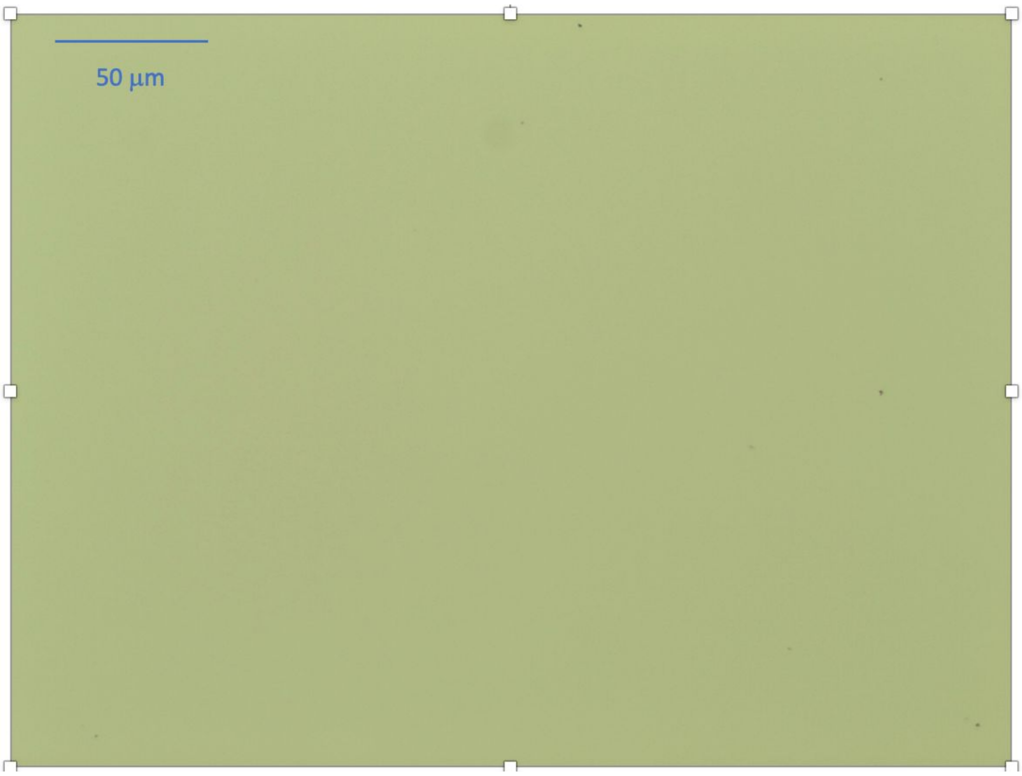

(b) 100 nm deposited film

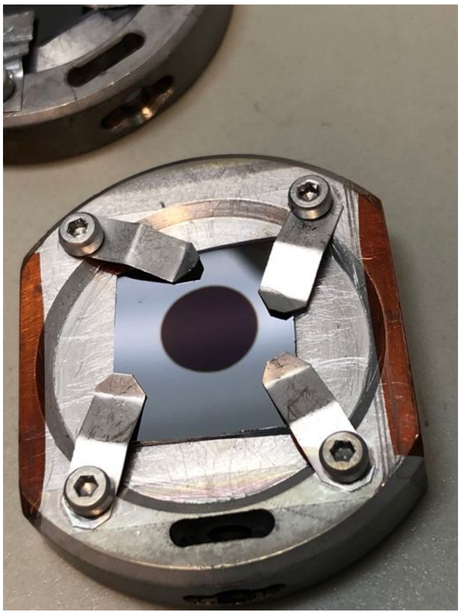

Optical microscope image of 100 nm sample position 1

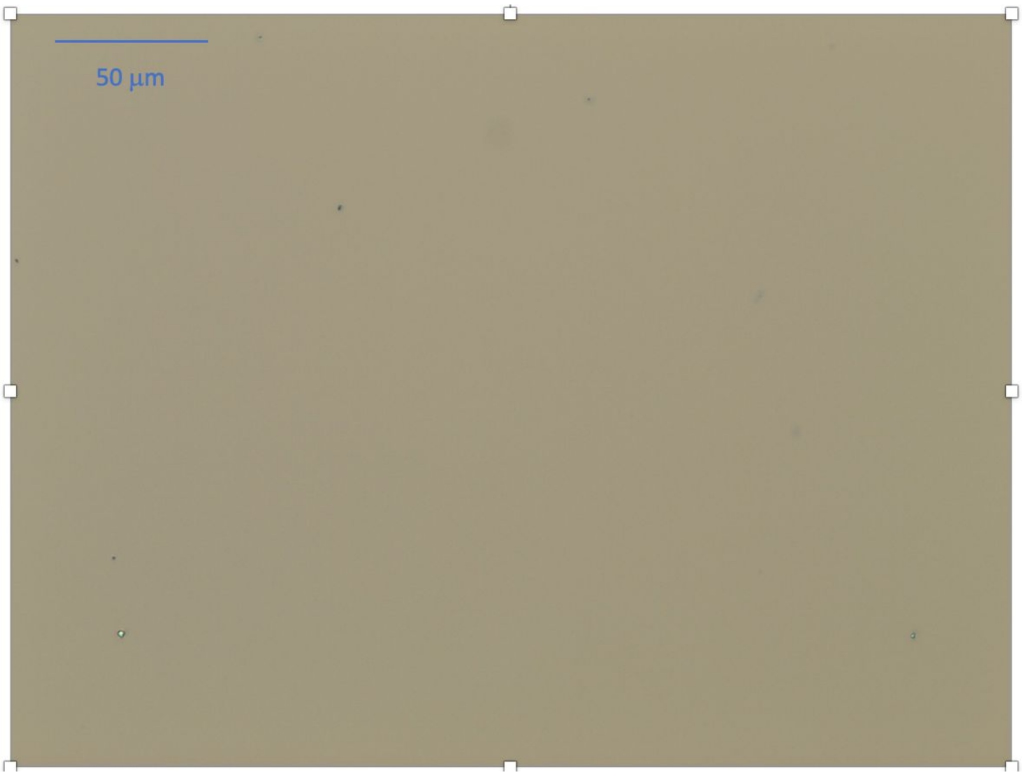

Optical microscope image of 100 nm sample position 2

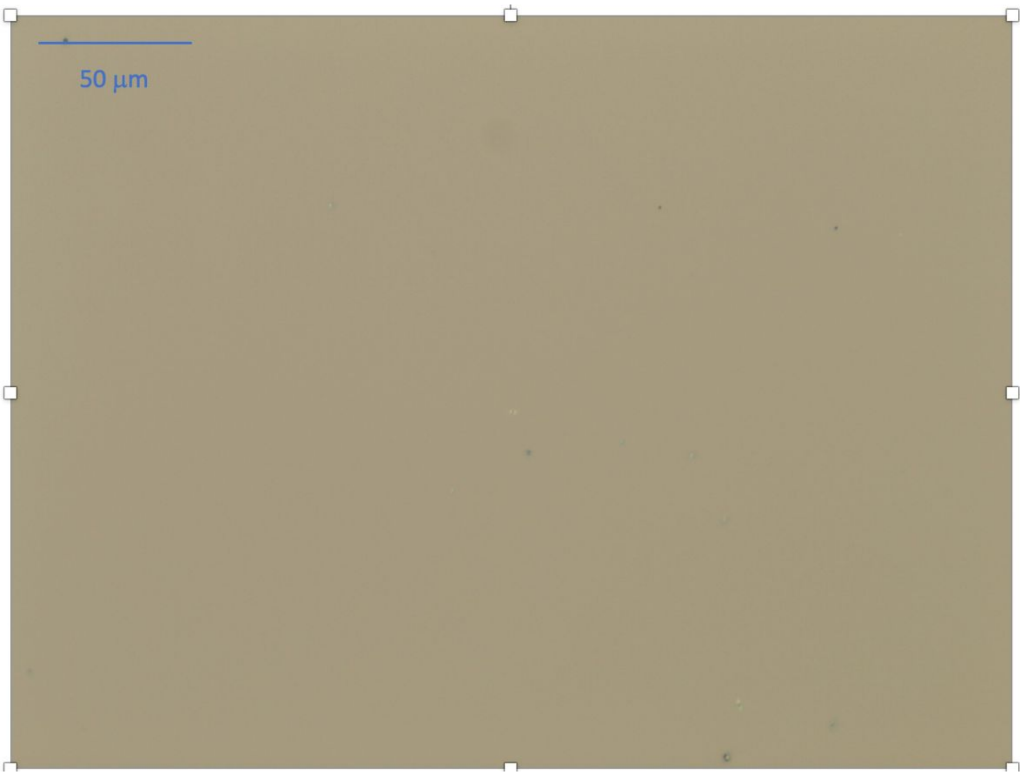

Optical microscope image of 100 nm sample position 3

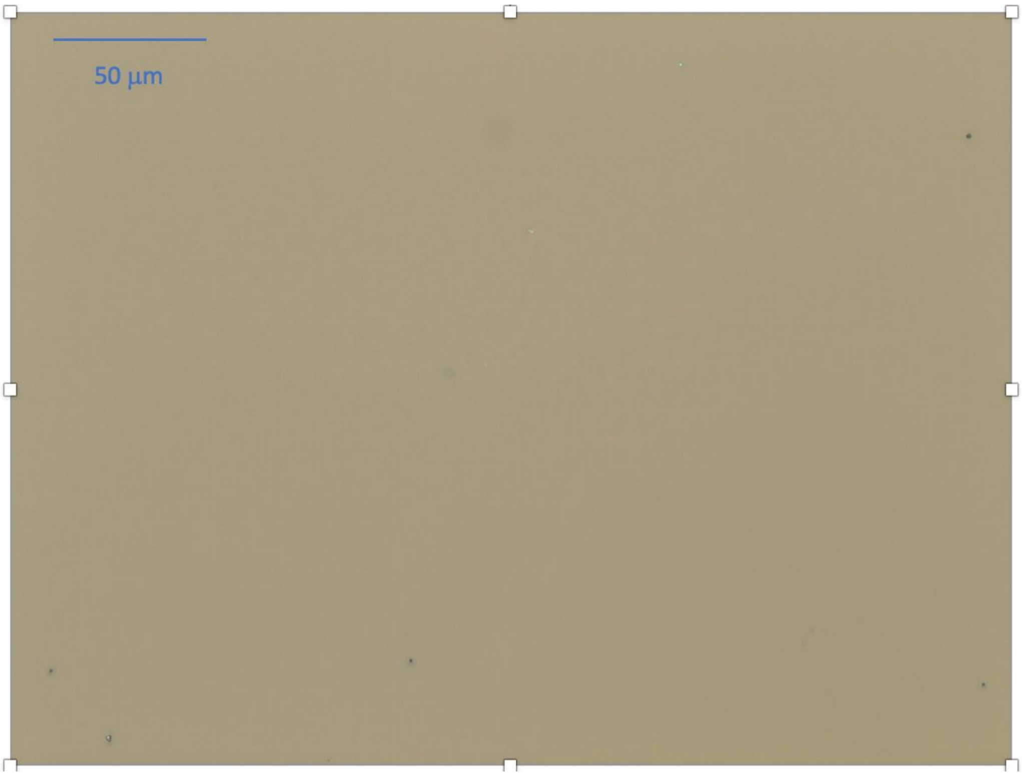

(c) 200 nm deposited film

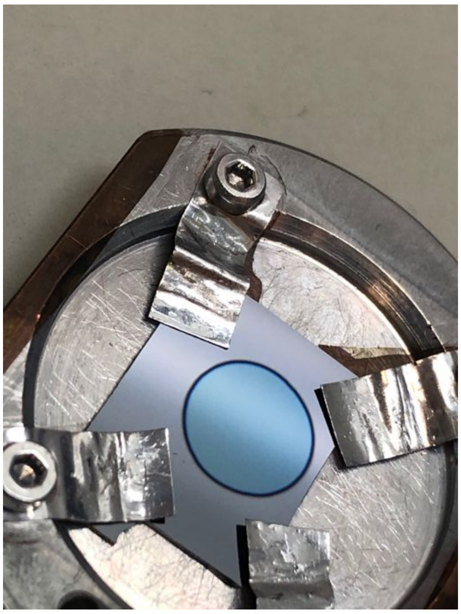

Optical microscope image of 200 nm sample position 1

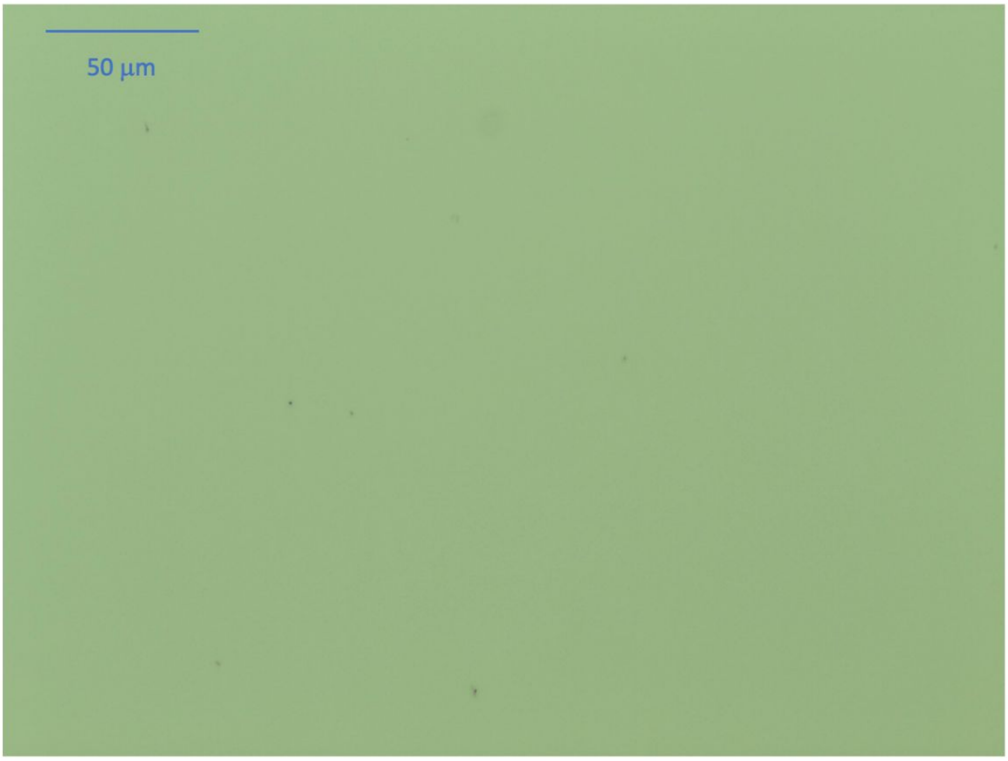

Optical microscope image of 200 nm sample position 2

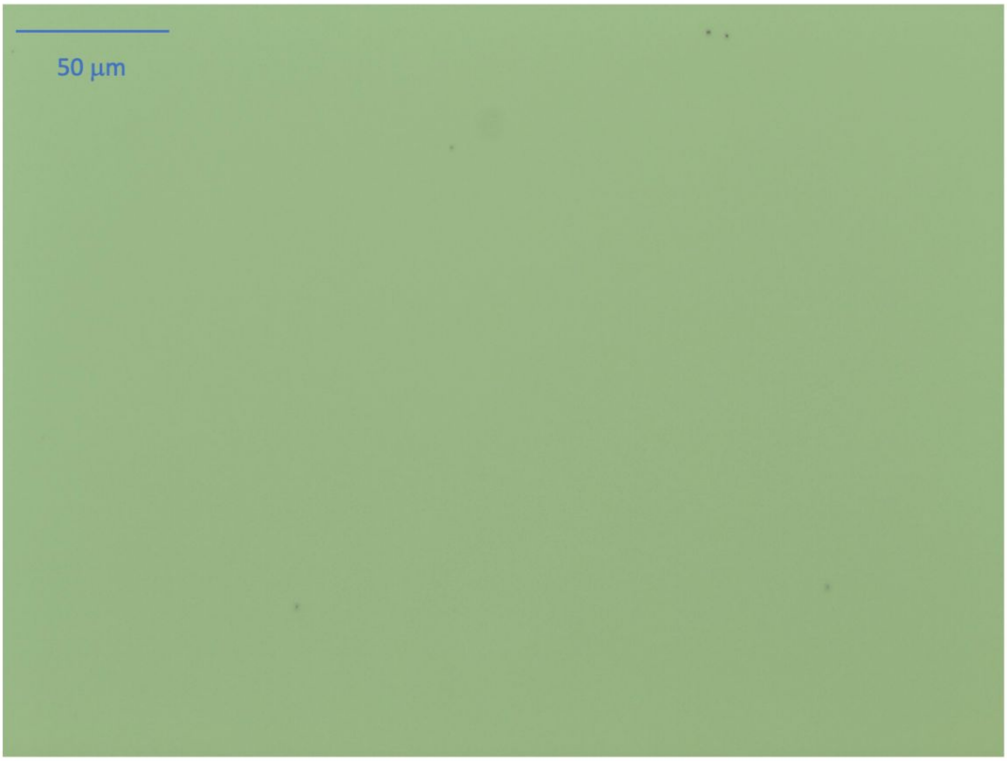

Optical microscope image of 200 nm sample position 3

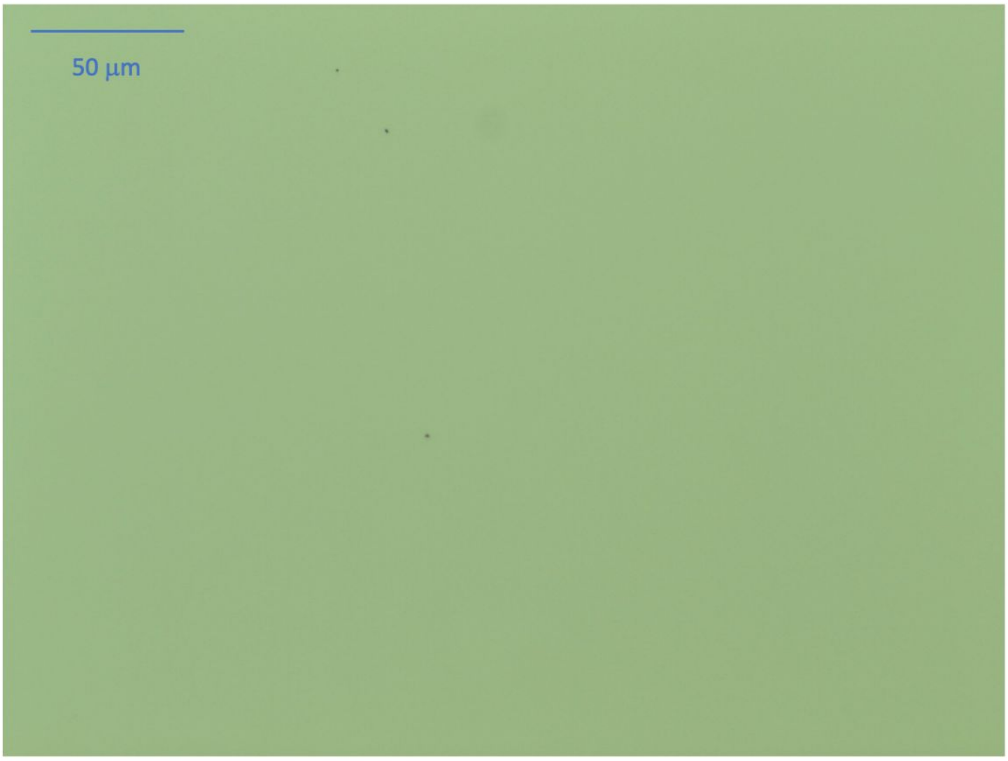

Optical microscope images of vapor-deposited CXB films of various thickness upon crystallization (taken after 6 months)

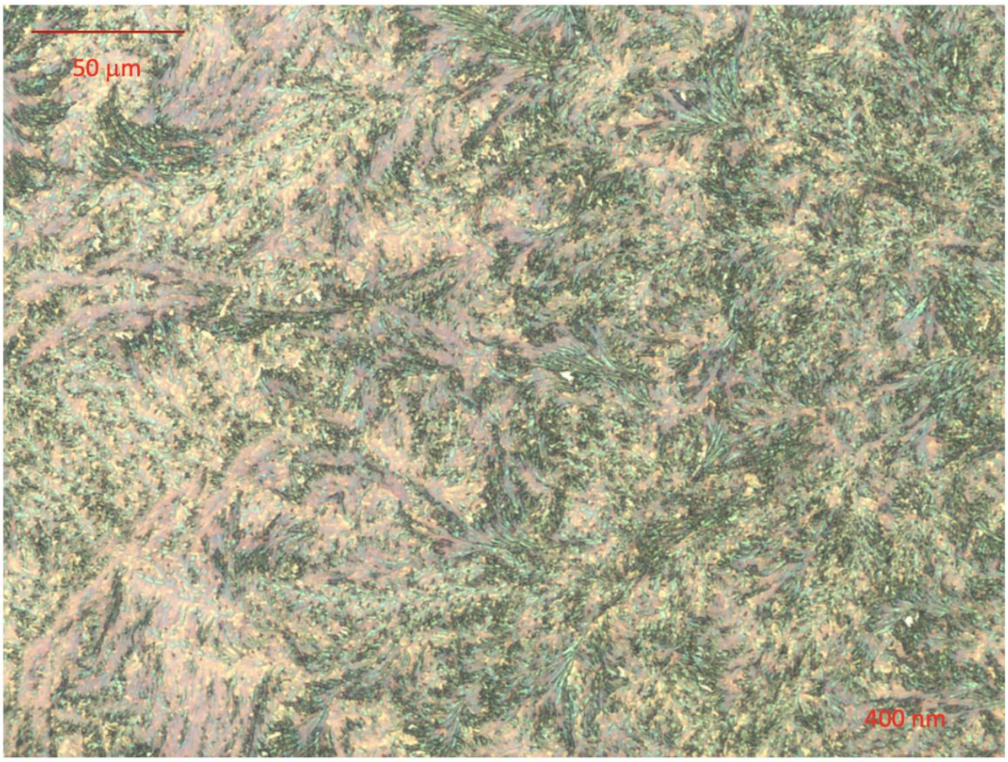

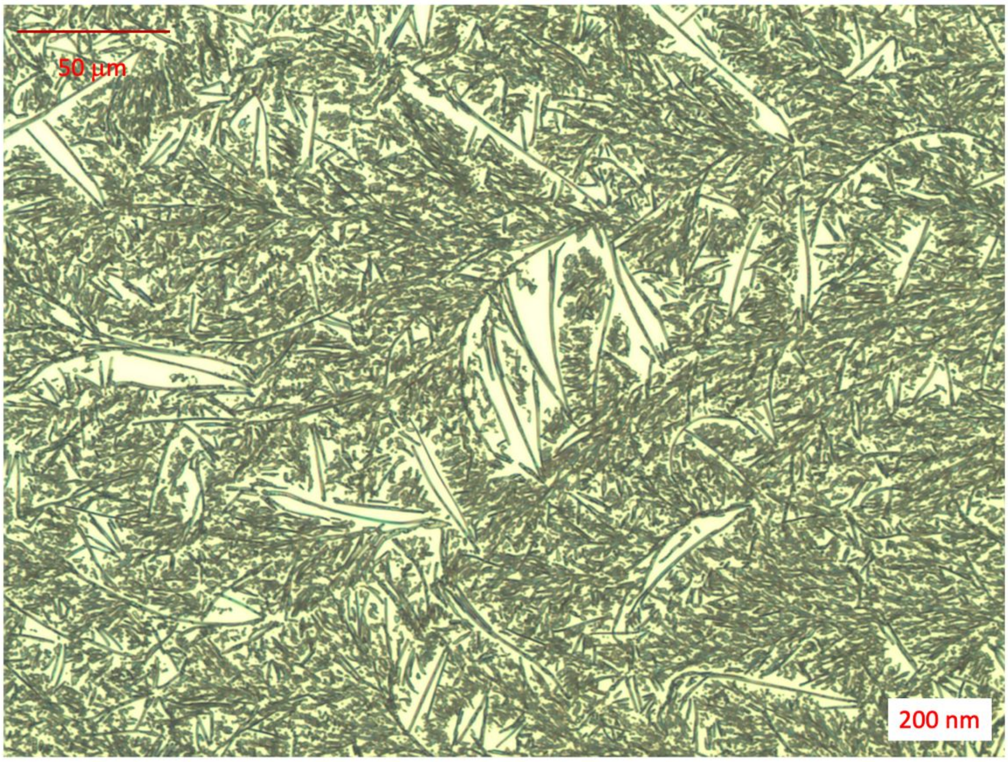
50 μm
200 nm

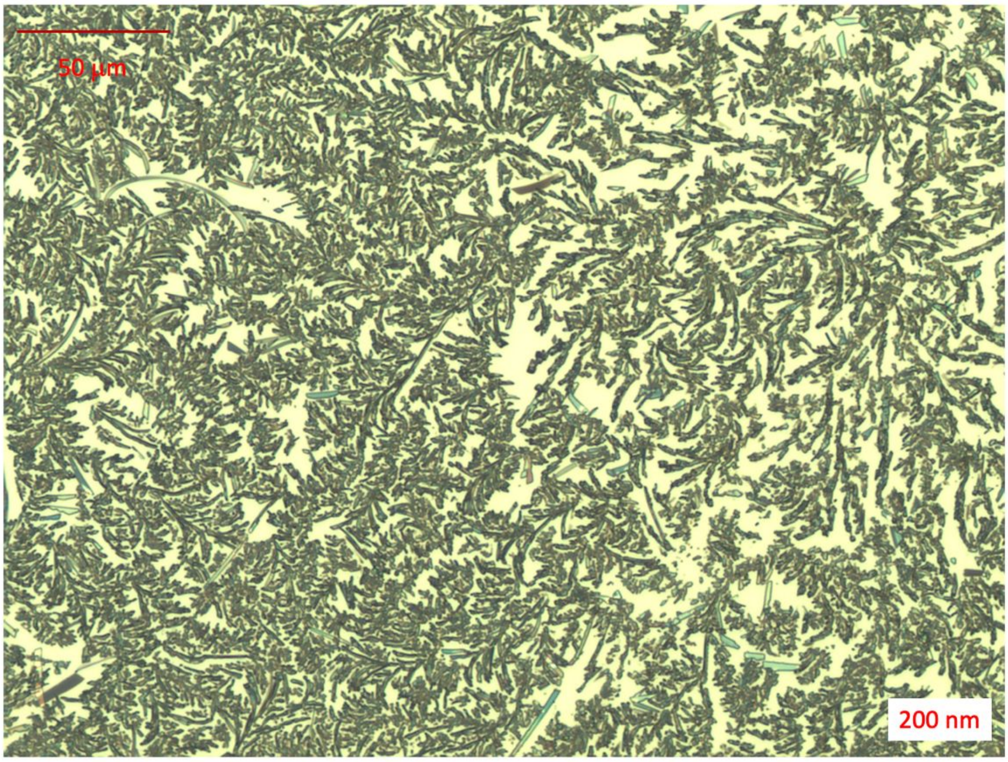
50 μm
200 nm

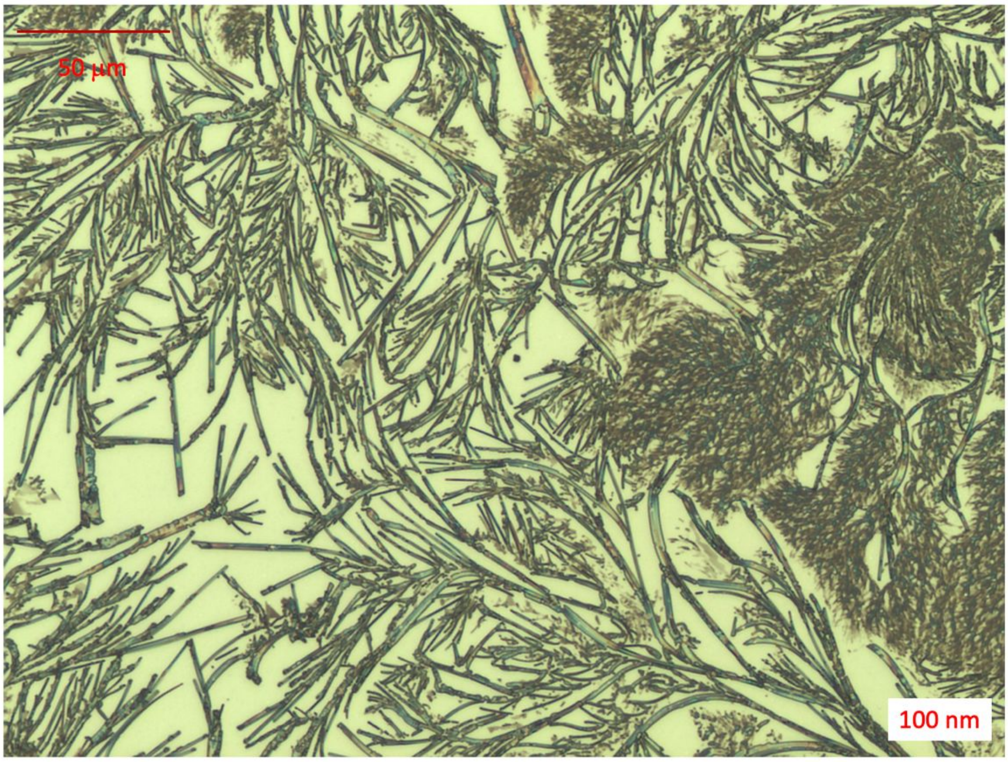
50 µm
100 nm

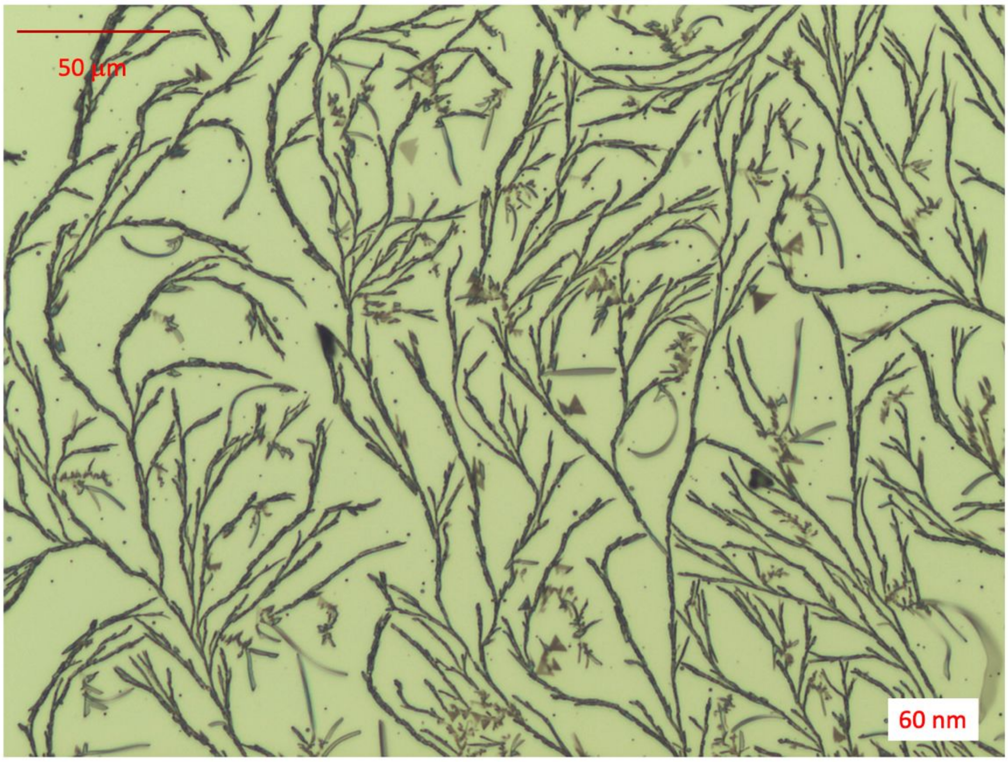
50 µm
60 nm

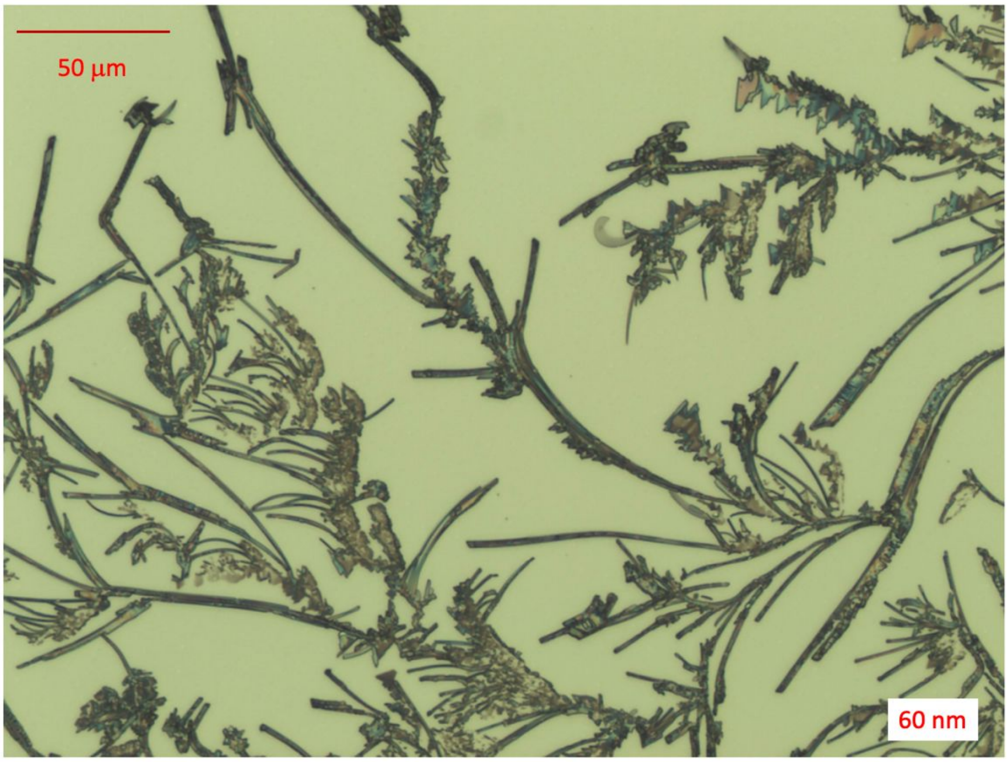
50 μm
60 nm

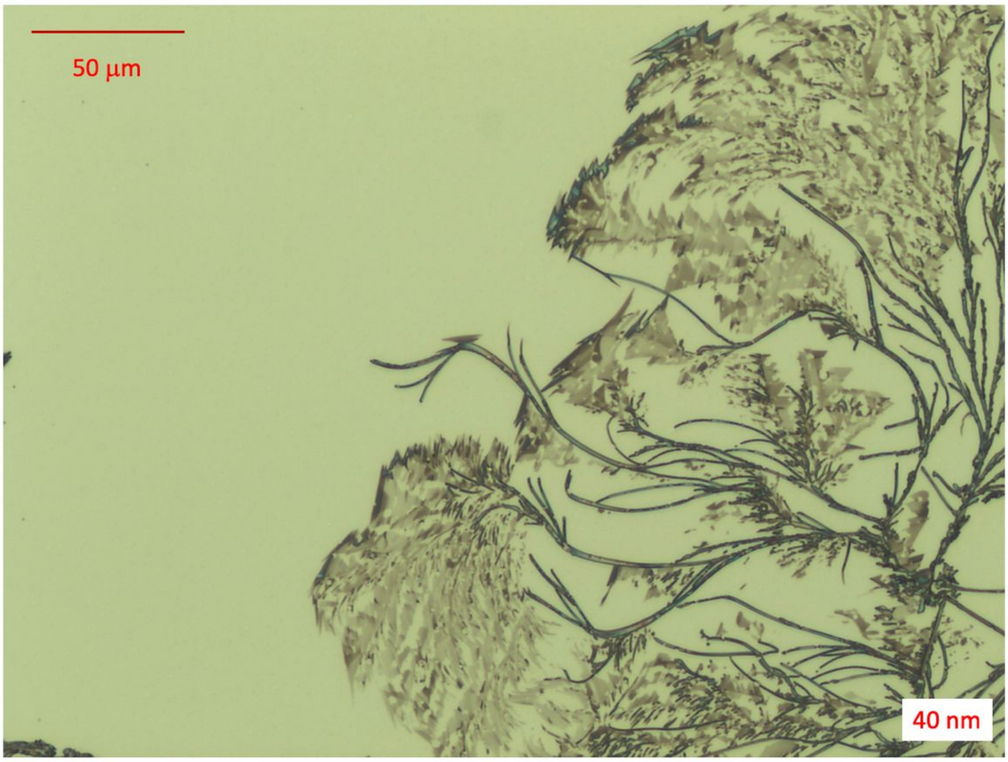
50 μm
40 nm

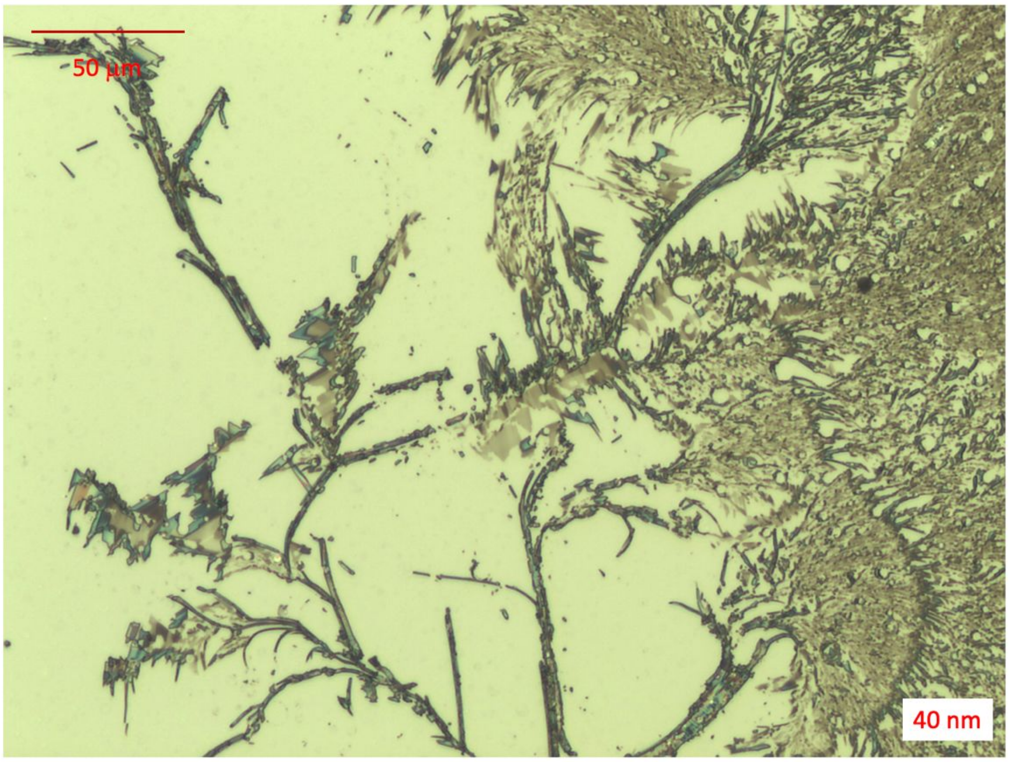
50 μm
40 nm

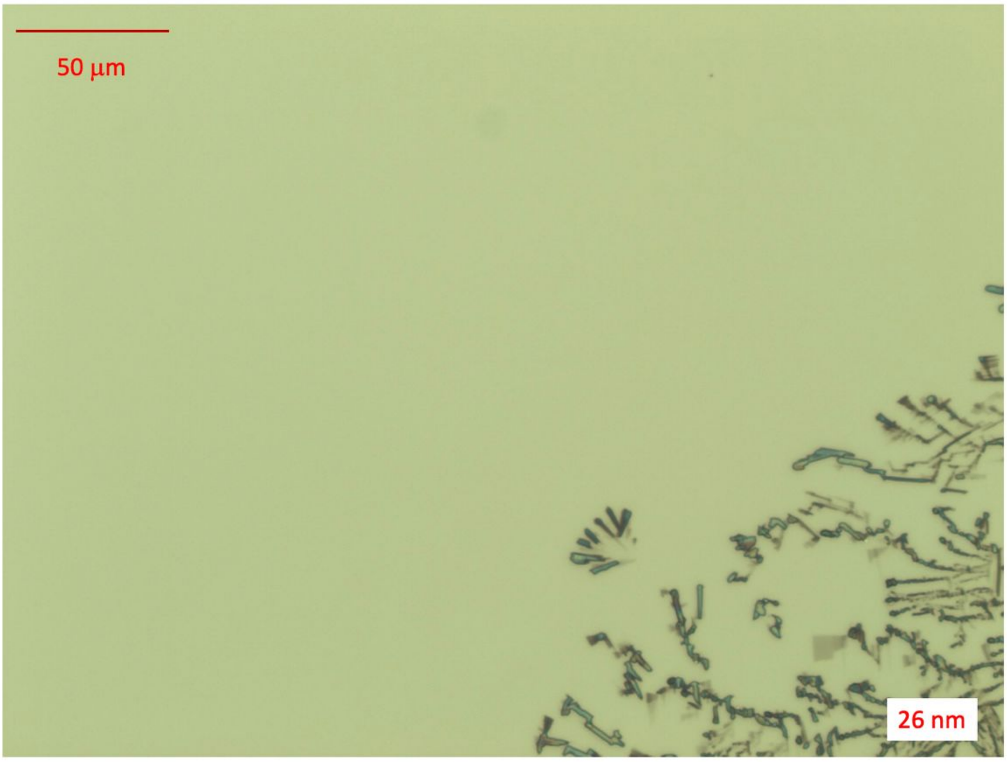
50 μm
26 nm

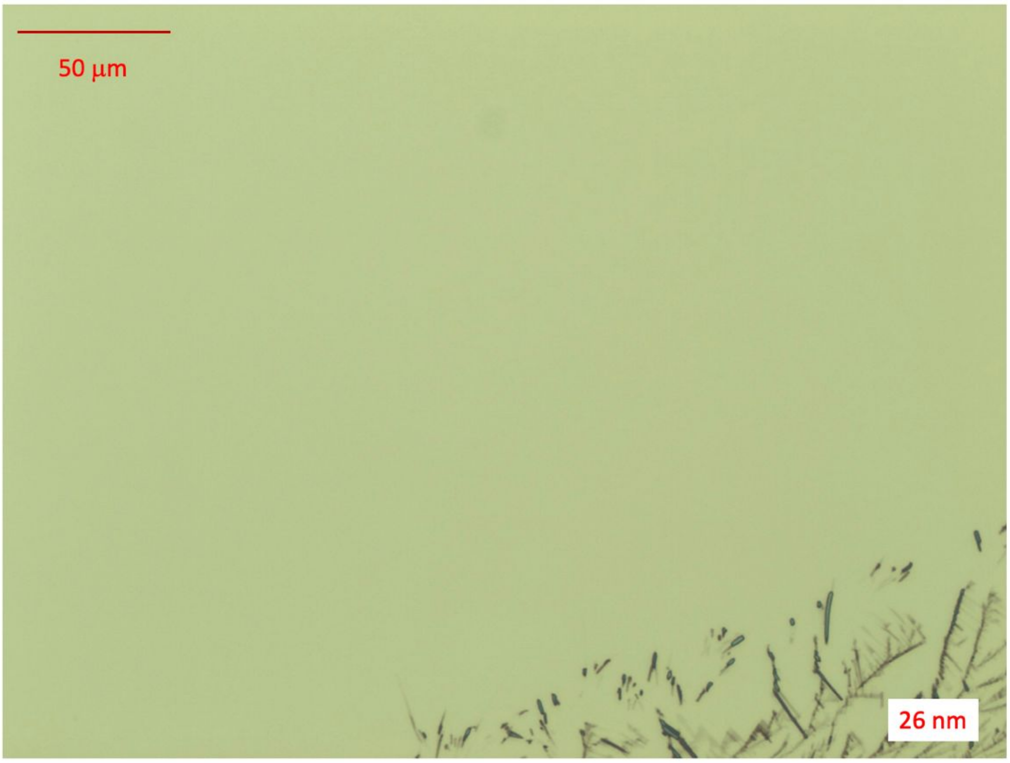
50 μm
26 nm

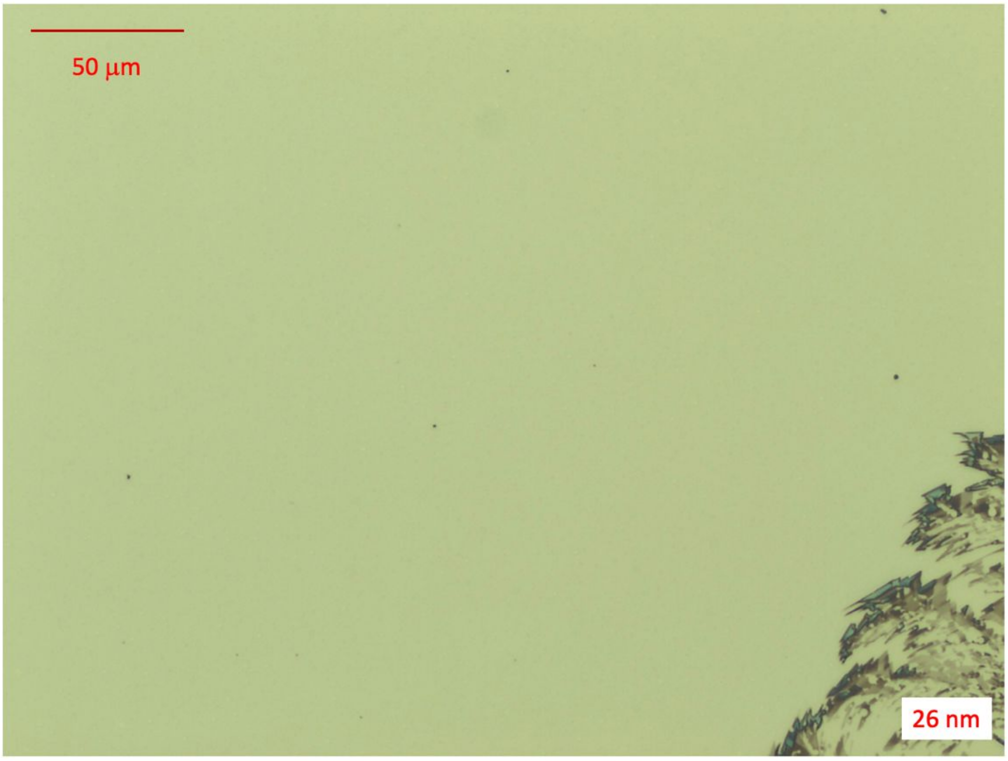
50 μm
26 nm

Figure S11. **AFM surface topography of 30 nm vapor deposited of celecoxib (CXB) film before measurements.**

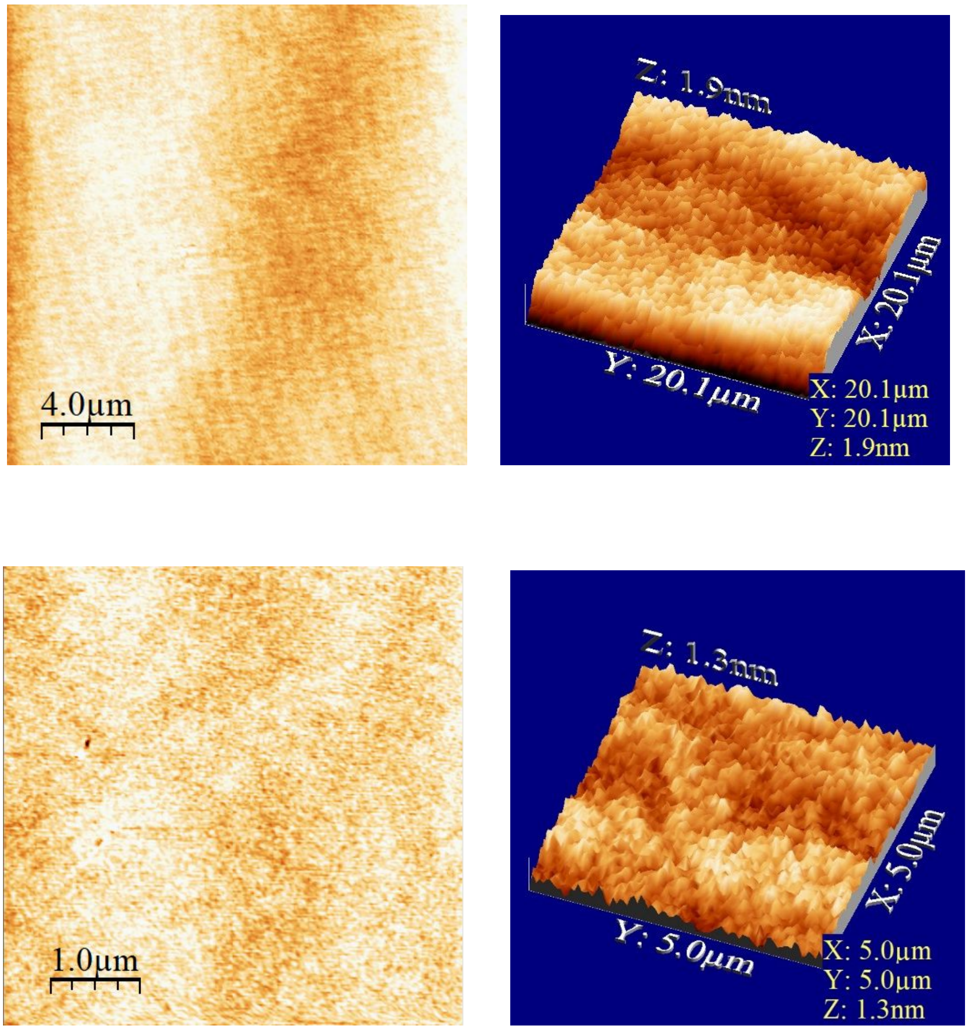

Figure S12. **AFM surface topography of 30 nm vapor deposited of celecoxib (CXB) film heated to the maximum measured temperature (100^{0}C)**

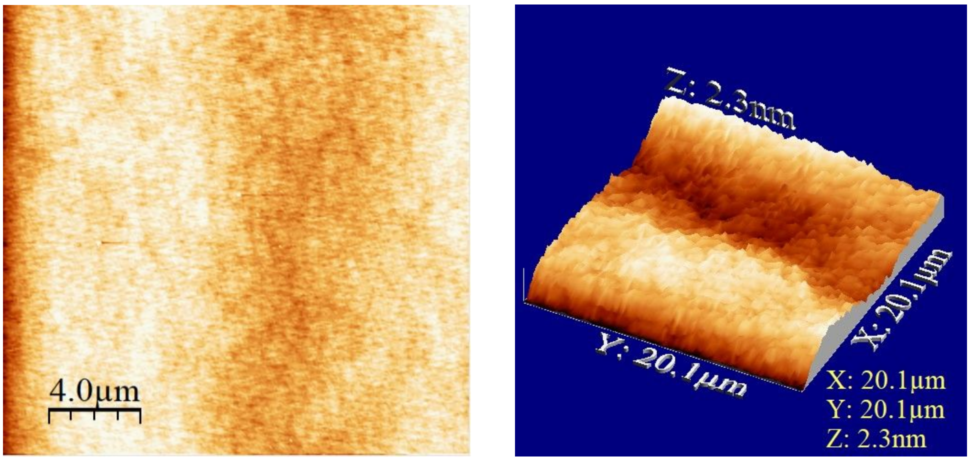

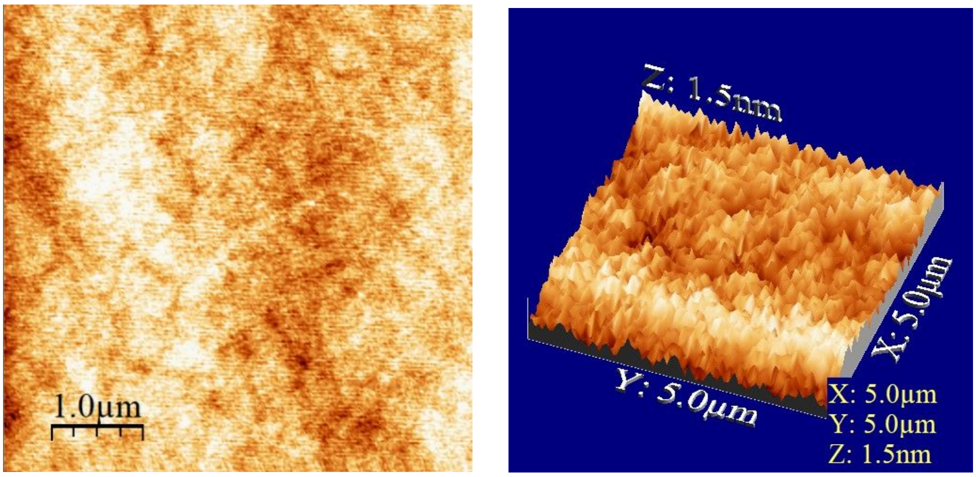

AFM surface topography of 30 nm vapor deposited of celecoxib before and after heating to the maximum temperature of the dielectric measurement demonstrates that the films do not dewett. Dewetting was also not observed in AFM scans for 30 nm thick vapor deposited films heated to 95°C (this was our selected crystallization temperature). Optical microscope images taken after AFM measurements confirmed that the samples do not dewett either. Hence the dewetting should not influence the reliability of our results.

Figure S13. **AFM surface topography of vapor deposited of celecoxib (CXB) film after the crystallization experiments done at 95^{0}C**

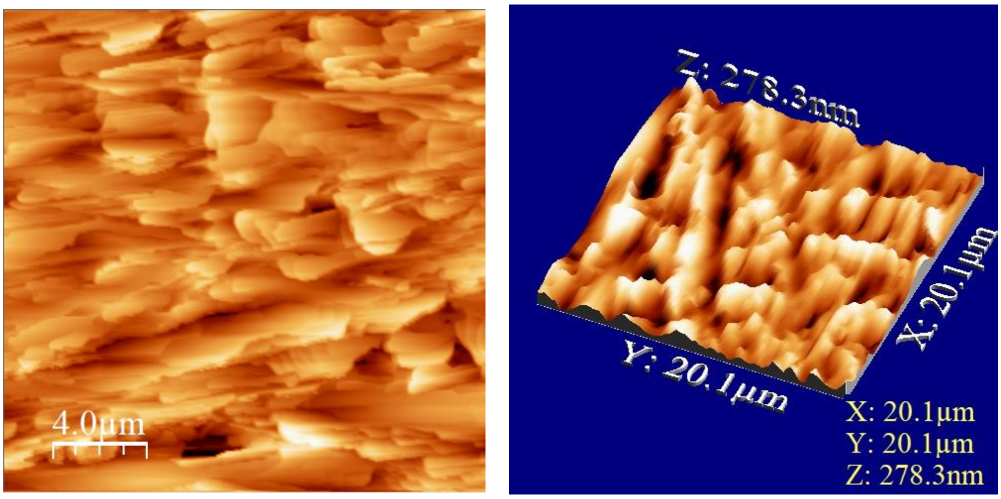

AFM images shown above demonstrates 30nm-film topology taken after the crystallization experiments. Crystallization was done above the T_g. In the case of complete film dewetting, we will not observe such a structuration throughout the film after the measurements.

Sample Dielectric loss spectra

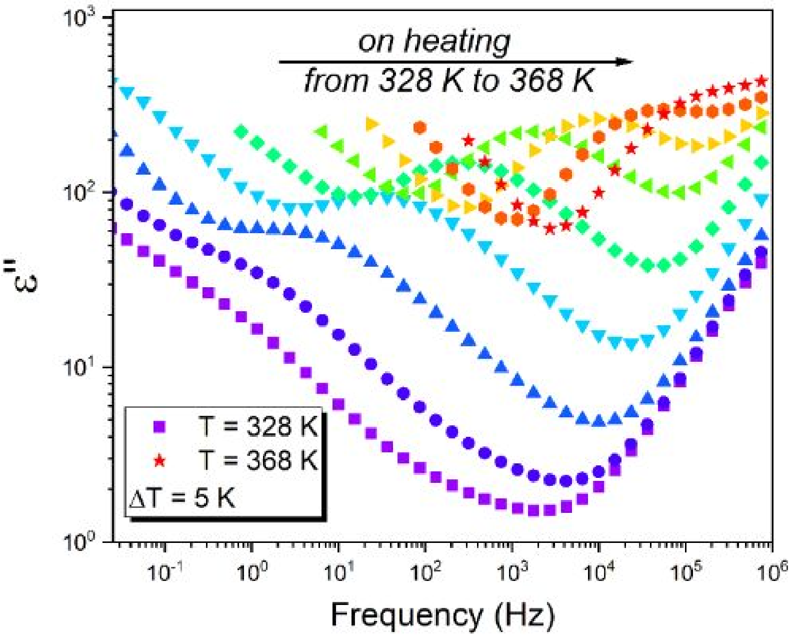

Figure S14. Dielectric loss spectra of 70 nm thick sample measured upon heating in the temperature range 328 K to 398 K.

References

[S1] H. B. Yu, M. Tylinski, A. Guiseppi-Elie, M. D. Ediger, and R. Richert. Suppression of β Relaxation in Vapor-Deposited Ultrastable Glasses. Phys. Rev. Lett. 115, 185501 (2015)

[S2] B. J. Kasting, M. S. Beasley, Guiseppi-Elie, R. Richert, and M. D. Ediger. Relationship between aged and vapor-deposited organic glasses: Secondary relaxations in methyl-m-toluate. J. Chem. Phys. 151, 144502 (2019)

[S3] M. S. Beasley, B. J. Kasting, M. E. Tracy, A. Guiseppi-Elie, R. Richert, and M. D. Ediger. Physical vapor deposition of a polyamorphic system: Triphenyl phosphite. J. Chem. Phys. 153, 124511 (2020)
